# Mortality risk according to smoking trajectories after cancer diagnosis among Korean male cancer survivors: A population-based cohort study

**DOI:** 10.18332/tid/163175

**Published:** 2023-05-25

**Authors:** Thi Tra Bui, Minji Han, Ngoc Minh Luu, Thi Phuong Thao Tran, Sun Young Kim, Young Ae Kim, Min Kyung Lim, Jin-Kyoung Oh

**Affiliations:** 1 Department of Cancer Control and Population Health, National Cancer Center Graduate School of Cancer Science and Policy, Goyang, Republic of Korea; 2 Department of Health Science and Technology, Graduate School of Convergence Science and Technology, Seoul National University, Seoul, Republic of Korea; 3 Center for Population Health Sciences, Hanoi University of Public Health, Hanoi, Vietnam; 4 Department of Cancer AI and Digital Health, National Cancer Control Institute, National Cancer Center, Goyang, Republic of Korea; 5 Cancer Survivorship Branch, National Cancer Control Institute, National Cancer Center, Goyang, Republic of Korea; 6 Department of Social and Preventive Medicine, College of Medicine, Inha University, Incheon, Republic of Korea; 7 Division of Cancer Prevention, National Cancer Control Institute, National Cancer Center, Goyang, Republic of Korea

**Keywords:** cancer, smoking, trajectory, cohort, survivor

## Abstract

**INTRODUCTION:**

Previous research on post-diagnosis smoking among cancer survivors mainly relied on smoking status, which may not fully reflect the impact of changes in smoking levels. This study aimed to evaluate mortality risk according to smoking trajectories among Korean male cancer survivors, using a trajectory approach to comprehensively capture smoking patterns.

**METHODS:**

The study included 110555 men diagnosed with cancer between 2002 and 2018 from the Korean National Health Information Database. Group-based trajectory modelling was used to identify post-diagnosis smoking trajectories among pre-diagnosis current smokers (n=45331). Cox hazards models were fitted to evaluate mortality risk according to smoking trajectories for pooled cancers, pooled smoking-related cancers, smoking-unrelated cancers, and gastric, colorectal, liver, and lung cancers.

**RESULTS:**

Smoking trajectories included light-smoking quitters, heavy-smoking quitters, consistent moderate smokers, and decreasing heavy smokers. Smoking significantly increased all-cause and cancer mortality risks in cancer patients for pooled cancers, pooled smoking-related cancers, and pooled smoking-unrelated cancers. Compared to non-smokers, all-cause mortality risk for pooled cancers significantly increased according to smoking trajectories:

(AHR=1.33; 95% CI: 1.27–1.40), (AHR=1.39; 95% CI: 1.34–1.44), (AHR=1.44; 95% CI: 1.34–1.54), and (AHR=1.47; 95% CI: 1.36–1.60), respectively. Smoking increased all-cause and cancer mortality risks in gastric and colorectal cancer patients and cancer-specific mortality in lung cancer patients. The significant associations of smoking trajectories with all-cause and cancer mortality risks were primarily observed in 5-year survivors but not in short-term survivors. Among heavy smokers, smoking cessation significantly reduced all-cause mortality risk in the long-term.

**CONCLUSIONS:**

The post-diagnosis smoking trajectory independently predicts cancer prognosis among male cancer patients. Proactive cessation support should be strengthened, particularly for those who smoke heavily.

## INTRODUCTION

The smoking epidemic is one of the biggest threats to public health. According to GLOBOCAN 2020, 8 out of 10 leading cancers worldwide among men are related to smoking in terms of cancer incidence and mortality^1^. In South Korea, where cigarette smoking remains high among men (36.7%), it was responsible for 20.9% of new cancer cases and 32.9% of cancer deaths in this population^2^. Smoking-related cancer burden measured in DALYs was estimated at 75117 person-years among men aged ≥40 years^3^. Notably, while tobacco use is a well-known risk factor for health, up to 38–52% of male cancer patients who smoke continue smoking after diagnosis^4,5^. The associations of smoking with the prognosis of cancer patients have been suggested in previous studies^4,6^. With medical advances and an ageing population, a foreseen increasing number of cancer survivors can result in an even larger tobacco-induced health burden.

A meta-analysis on prostate cancer reported poor prognosis in terms of overall survival, cancer-specific survival, and recurrence-free survival associated with smoking after cancer diagnosis compared with those for never smokers^7^. Among patients with head and neck cancer treated with radiotherapy, those who continued smoking have a significantly higher risk of locoregional failure, almost twofold, than those who quit smoking^8^. It has been suggested that smoking may increase the risk of surgical complications^9^, reducing treatment response^10^. Tobacco nitrosamines have been shown to stimulate cancer cell proliferation, suppress apoptosis, induce angiogenesis, and facilitate cell migration, thus accelerating cancer progression and metastasis^11,12^. Moreover, smoking may also lead to interactions and adverse effects in and post-cancer treatment and exacerbate comorbidities that negatively impact patients’ quality of life^13^. Nevertheless, the underlying mechanisms of the association between smoking and cancer prognosis are yet to be fully understood, raising the need for further prospective studies on the impact of post-diagnosis smoking on cancer progression^7,14,15^. Notably, the simultaneous investigation of different cancer types may be helpful to elaborate the findings from studies on specific cancer types.

On the other hand, changes in smoking behavior may lead to changes in cancer prognosis. Smoking cessation among colorectal cancer survivors showed a clear improvement of 22% in overall survival in both the short-term (<10 years) and long-term (≥10 years), and 24% in cancer-specific survival in the long-term^6^.

However, it should be noted that previous studies mainly relied on smoking status to inform behavior changes in cancer patients ^4-6, 8, 14, 16, 17^, which may not fully reflect the impacts of changes in smoking levels. For instance, the classification of smoking behavior may include three categories of smoking status at diagnosis (current, ex-smokers, and never smokers)^14,17^, or four categories of pre- and post-diagnosis smoking status combined (non-smokers/non-smokers, non-smokers/smokers, smokers/non-smokers, and smokers/smokers)^4^. As more evidence is needed to emphasize the importance of smoking cessation in cancer survivorship care^18^, it is meaningful to investigate smoking patterns after cancer diagnosis comprehensively. Trajectory analysis^19^ is a method that has recently been adopted in epidemiology to track temporal behavior patterns^20^. To the best of our knowledge, no studies have ascertained smoking trajectories after a cancer diagnosis to date. Therefore, this study aimed to evaluate the association between smoking and mortality risk among South Korean male cancer survivors using trajectory analysis to comprehensively capture smoking patterns after a cancer diagnosis.

## METHODS

### Data source and study population

The National Health Information Database (NHID) is a public database of the National Health Insurance Service (NHIS) that covers the whole population of South Korea (>50 million people)^21^. It includes annually updated data on sociodemographics, healthcare utilization, mortality, and biennially collected health screening data for insured participants and their dependents^21^.

This is a population-based cohort study. We used a customized NHIS database of 8968212 individuals who attended the 2002–2003 general health examination and were followed up to 2018. Among 805430 incident cancer cases, we excluded 326567 women due to a low smoking rate (4.2–6.5%)^16,22^. We further excluded 63864 participants aged <40 or >79 years because individuals who are younger than 40 years have a substantially lower chance of getting cancer related to unhealthy lifestyles, and those aged ≥80 years might already have poorer health conditions that smoking might affect their survival with a different pattern from those in the adult population. After excluding 199137 participants missing data on smoking behavior within six years before or six years after cancer diagnosis and 4 cases with an invalid death date, the study finally included 110555 male cancer patients (flowchart of patient selection, Supplementary file Figure S1).

### Exposure and covariates

*Exposure*

Data on smoking behavior were collected using a self-administered questionnaire containing information on smoking status (never, former, and current smokers) and daily amount of smoking (cigarettes/day). There were six smoking levels: 1) never smokers, 2) former smokers, 3) current smokers, 1–10 cigarettes/day; 4) current smokers, 11–20 cigarettes/day; 5) current smokers, 21–40 cigarettes/day; and 6) current smokers, >40 cigarettes/day. Data on smoking levels were obtained twice: pre-diagnosis (within six years before cancer diagnosis) and post-diagnosis (within six years after cancer diagnosis). The 6-year period was decided, considering the trade-off between the heterogeneity of the study population regarding smoking measurement time-point and the sample size, which is also related to the magnitude of selection bias. In the case of multiple available measurements, the highest pre-diagnosis smoking level and the latest post-diagnosis measurement were selected. Trajectory analysis was conducted only on pre-diagnosis current smokers, given that smoking initiation after diagnosis is not common among pre-diagnosis never and former smokers^4^. As the trajectory analysis requires at least three measurements, the third smoking measurement was imputed from the post-diagnosis measurement. Smoking patterns after cancer diagnosis were interpreted based on the first two measurements. To examine the robustness of our main analysis findings, we additionally examined the sub-population with three smoking measurements: pre-diagnosis measurement, early post-diagnosis measurement (i.e. at 2 years post-diagnosis), and late post-diagnosis measurement (at 2–6 years post-diagnosis).

For comparison with the trajectory approach, we included two conventional methods of smoking classification. Separate analyses were performed for smoking groups defined by trajectory analysis, smoking status, and baseline smoking levels. In the status-based smoking classification, there were four categories: non-smokers (pre-diagnosis non-smokers), former smokers (pre-diagnosis former smokers), smoking quitters (pre-diagnosis current smokers who quit after diagnosis), and continued smokers (pre-diagnosis current smokers who continued smoking after diagnosis).

*Covariates*

The covariates included age at cancer diagnosis (continuous), household income (quintiles), body mass index (BMI) (<18.5, 18.5–22.4, 22.5–24.9, and ≥25 kg/m^2^), alcohol consumption frequency (0, 1–2, 3–4, and ≥5 times/week), physical activity frequency (0–2 and ≥3 days/week), and Charlson comorbidity index (CCI) (continuous)^23,24^. Those covariates were selected to include in Cox hazards models to adjust for confounding effects based on biological plausibility^4^. Data on covariates were extracted at baseline (2002–2003), except for CCI, obtained within two years before cancer diagnosis.

### Case ascertainment

Cancer cases were those with a primary cancer diagnosis (defined by ICD-10 codes C00–C97) and further confirmed by ‘V193’, a special code introduced in 2005 to expand insurance benefits for patients with cancer. The date of cancer diagnosis was defined as the first date of diagnosis with C code. The events of interest included all-cause, cancer, and cancer-specific deaths. We assessed the association between smoking and the risk of these outcomes for pooled cancers of all types, pooled smoking-related cancers, pooled cancers not related to smoking, and four leading cancer types in terms of cancer incidence and mortality in Korean men (2020): gastric, colorectal, liver, and lung cancers^1^. Smoking-related cancers include cancers of the lip, oral cavity, pharynx, esophagus, stomach, colorectum, liver, pancreas, larynx, lung, kidney, bladder, and leukaemia^25^. Cancer types other than smoking-related cancers were pooled into the group of cancers unrelated to smoking.

### Statistical methods

*Descriptive statistics*

Data for continuous variables (age at baseline, age at cancer diagnosis, and survival time) are presented as mean with standard deviation (SD). The frequency and percentage are used for categorical variables (age, BMI, alcohol consumption, physical activity, CCI, and smoking classification).

*Trajectory analysis*

Group-based trajectory modeling was used to identify smoking patterns after cancer diagnosis^19^. This is a semi-parametric finite mixture modeling approach^19,20^. Among different trajectory analysis methods commonly used in epidemiology, we adopted this method because it produces less-complex models with more straightforward interpretation and requires less computing time^20^. We fitted the censored normal model for the smoking level presented in ordinal categories using PROC TRAJ procedure installed in SAS 9.4 (SAS Institute Inc., Cary, NC, USA)^19,26^.

Model selection

**Figure 1:**
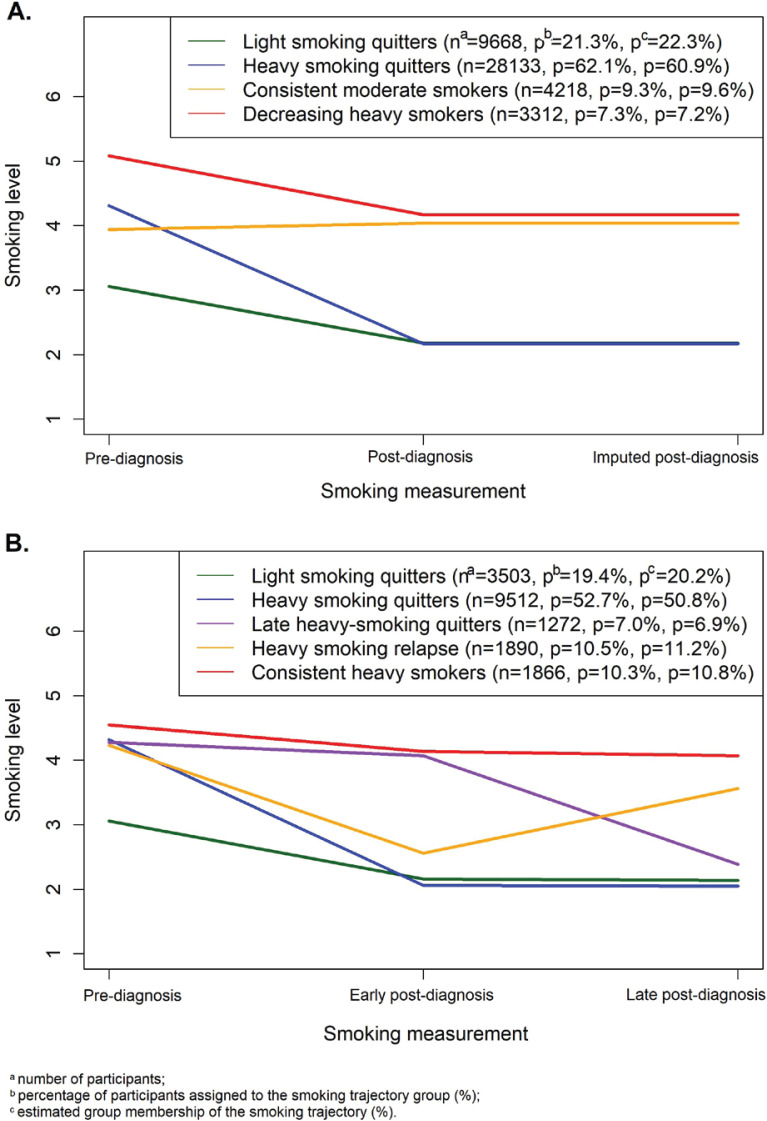
Smoking trajectories of pre-diagnosis current smokers during cancer diagnosis: A) Model (2,2,2,2) for the entire study population (n=45331); and B) Model (2,2,2,2,2) for the three-measurement subpopulation (n=18043)

We decided on the maximum group number of 7 because the selected models in previous studies on smoking trajectories consisted of no more than seven groups^27,28^. We fitted regression models with group numbers from 1 to 7, and all groups in quadratic order. As the Bayesian information criterion (BIC) values increased with additional groups, we decided on the optimal group number based on group membership (≥5%), model parsimony, and the models’ ability to present distinct features of interest (i.e. to differentiate smoking patterns according to pre-diagnosis smoking levels). As a result, a model with four groups was selected because of its ability to separate smoking levels at pre-diagnosis (light/moderate/heavy) and differentiate smoking patterns at post-diagnosis (quitting/continuing). To optimize the group order, the non-significant quadratic order (p<0.05) was reduced to linear^26^. The (2,2,2,2) model, with the most significant BIC score (negative) among the tested models, was selected (Figure 1A). For the three-measurement subpopulation, the (2,2,2,2,2) model was selected as the best-fit model (Figure 1B) (see Supplementary file Table S1 for the model selection process).

Model evaluation

The two selected models were evaluated using the average posterior probability of assignment (AvePP) and odds of correct classification (OCC). Despite no definite cut-off values for these diagnostic criteria, AvePP ≥0.7 and OCC ≥5.0 were recommended^19^. The selected models showed high AvePP (≥0.94) and OCC (≥19.9) values in all groups; therefore, they were considered acceptable for further analysis (Supplemental Table S2).

Details of smoking trajectories

For the entire study population (n=110555), smoking trajectories for pre-diagnosis current smokers (n=45331; 41.0%) included: T1.1) light smoking quitters (n=9668; 21.3% of pre-diagnosis current smokers and 8.7% of the study population); T1.2) heavy smoking quitters (n=28133; 62.1%, and 25.4%, respectively); T1.3) consistent moderate smokers (n=4218; 9.3%, and 3.8%, respectively); and T1.4) decreasing heavy smokers (i.e. heavy smokers who decrease smoking level) (n=3312; 7.3%, and 3.0%, respectively). The average duration from the time of measurement to cancer diagnosis was 4.0 ± 1.7 years for pre-diagnosis measurement and 4.4 ± 1.5 years for post-diagnosis measurement. For the three-measurement subpopulation (n=43401), the prevalence of smoking at pre-diagnosis was 41.6% (n=18043). The five smoking trajectories among pre-diagnosis current smokers were: T2.1) light smoking quitters (n=3503, 19.4% of pre-diagnosis current smokers and 8.1% of the study population); T2.2) heavy smoking quitters (n=9512; 52.7%, and 21.9%, respectively); T2.3) late heavy smoking quitters (n=1272; 7.0%, and 2.9%, respectively); T2.4) heavy smoking relapse (n=1890; 10.5%, and 4.4%, respectively); and T2.5) consistent heavy smokers (n=1866; 10.3%, and 4.3%, respectively). The average duration from smoking measurement to cancer diagnosis was 4.2 (SD=1.7) years for the pre-diagnosis measurement, 2.0 (SD=0.1) years for the early post-diagnosis measurement, and 5.1 (SD=1.0) years for the late post-diagnosis measurement.

*Cox proportional hazard regression*

Cox proportional hazards regression was used to evaluate the association between smoking and mortality risk. Time-to-event (years) was estimated as the duration from the date of cancer diagnosis to death or the end of 2018, whichever occurred first. In the analysis of all-cause mortality, the censored cases were cancer survivors at the end of the study period. In the analysis of cancer mortality, censored cases included patients who died from causes other than cancer or survived until the end of 2018. Similarly, for cancer-specific mortality, participants who died of causes other than that specific cancer were censored on their death date, and those who survived at the end of 2018 were censored on 31 December 2018. The variable that indicates the trajectory group in which a participant was assigned was treated as a time-invariant covariate (categorical), considering that this variable reflects the smoking pattern over time. The group of non-smokers was used as the reference. All analyses were stratified according to the survival time (<5 and ≥5 years). A linear trend was assessed using the log-rank test, and a p<0.05 was considered significant. The Cox proportional hazards assumption was assessed with the Kaplan-Meier survival curves for categorical covariates and Schoenfeld residuals for continuous covariates, showing the assumption was satisfied for all covariates (refer to Supplemental Figure S2 for survival curves of smoking covariates).

We performed competing risk analyses based on the cause-specific hazard function, in which the likelihood function for an event of interest treats all other events as censored at their occurrence time^29^. This approach allows us to estimate the effect of covariates on the occurrence rate of the outcome among participants who are currently event-free^29^. Specifically, in the cancer mortality analysis, the competing risks included deaths from other causes. In the analysis of cancer-specific mortality, the competing risks were deaths due to other cancer types and causes. We additionally explored all-cause mortality risks according to smoking trajectories for some cancer types (GLOBOCAN cancer dictionary) other than the four cancer types investigated in the main analysis^30^. An alternative analysis was performed with decreasing heavy smokers (T1.4) as the reference to reveal the implications for behavioral change.

*Sensitivity analysis*

Mortality risks according to smoking trajectories were re-assessed with the Cox hazards regression models, including alcohol drinking, physical activity, and body mass index as time-dependent covariates. Data on those variables were collected on the same general health examinations from which we extracted data on smoking behavior. In the trajectory analysis for the entire study population, data imputation for the third measurement assumed participants retained their post-diagnosis smoking behavior throughout the post-diagnosis period. Therefore, we additionally performed trajectory analyses for pre-diagnosis current smokers who had multiple smoking measurements after diagnosis. Five subgroups (each contributing to 10% of the smoking population or above) were selected: participants with at least two, three, four, five, and six measurements after a cancer diagnosis.

## RESULTS

### General characteristics of the study population and cancer incidence

Study participants were aged, on average, 55.6 ± 8.8 years at baseline and 63.0 ± 8.8 years at their primary cancer diagnosis (Table 1). At the time of study entry, most participants were overweight or obese (66.3%), drank alcoholic beverages (64.1%), and did not engage in regular physical exercise (75.9%). Current smokers accounted for 41.0%, and most consumed ≥10 cigarettes/day. On average, patients lived for 8.3 ± 3.4 years after the primary cancer diagnosis. Regarding smoking trajectories, 16.6% of the pre-diagnosis current smokers (n=7530; 6.8% of the study population) continued smoking after a cancer diagnosis. In the three-measurement subpopulation (Supplementary file Table S3), among 82.6% of the pre-diagnosis current smokers (n=14905; 34.3% of the subpopulation) engaging in smoking cessation or reduction, 10.5% relapsed to smoking (n=1890; 4.4% of the subpopulation). Patients who tended to continue smoking, relapse, or delay in quitting were mostly pre-diagnosis heavy smokers who smoked 20 cigarettes/day or more.

**Table 1:** General characteristics of the study population at the study entry (2002–2003) (N=110555)

	*Study population (N=110555) n (%)*	*Non-smokers (N=24664) n (%)*	*Former smokers (N=40560) n (%)*	*T1.1 Light smoking quitters ^a^ (N=9668) n (%)*	*T1.2 Heavy smoking quitters ^a^ (N=28133) n (%)*	*T1.3 Consistent moderate smokers ^a^ (N=4218) n (%)*	*T1.4 Decreasing heavy smokers ^a^ (N=3312) n (%)*
**Age** (years), mean ± SD	55.6 ± 8.8	57.4 ± 8.9	56.4 ± 8.8	56.7 ± 9.1	53.5 ± 8.3	53 ± 8.3	51.1 ± 7.4
**Age group** (years)							
40–49	31433 (28.4)	5378 (21.8)	10221 (25.2)	2497 (25.8)	10098 (35.9)	1656 (39.3)	1583 (47.8)
50–59	39578 (35.8)	8230 (33.4)	14701 (36.2)	3066 (31.7)	10808 (38.4)	1547 (36.7)	1226 (37.0)
60–69	33235 (30.1)	9015 (36.6)	12967 (32.0)	3405 (35.2)	6489 (23.1)	895 (21.2)	464 (14.0)
70–79	6309 (5.7)	2041 (8.3)	2671 (6.6)	700 (7.2)	738 (2.6)	120 (2.8)	39 (1.2)
**Age at cancer diagnosis** (years), mean ± SD	63.0 ± 8.8	64.5 ± 8.8	64.2 ± 8.8	64 ± 9.0	60.9 ± 8.2	59.7 ± 8.1	57.9 ± 7.4
**Income** (quintile)							
1st	11345 (10.3)	2278 (9.2)	3922 (9.7)	1052 (10.9)	3184 (11.3)	508 (12.0)	401 (12.1)
2nd	12765 (11.5)	2663 (10.8)	4364 (10.8)	1225 (12.7)	3537 (12.6)	569 (13.5)	407 (12.3)
3rd	20548 (18.6)	4252 (17.2)	7306 (18.0)	1807 (18.7)	5576 (19.8)	895 (21.2)	712 (21.5)
4th	22586 (20.4)	4939 (20.0)	8229 (20.3)	1967 (20.3)	5817 (20.7)	904 (21.4)	730 (22.0)
5th	40805 (36.9)	10006 (40.7)	15845 (39.0)	3381 (35.0)	9328 (33.1)	1255 (29.8)	990 (29.9)
Missing	2506 (2.3)	526 (2.1)	894 (2.2)	236 (2.4)	691 (2.5)	87 (2.1)	72 (2.2)
**BMI** (kg/m^2^)							
<18.5	2015 (1.8)	337 (1.4)	560 (1.4)	290 (3.0)	642 (2.3)	110 (2.6)	76 (2.3)
18.5–22.4	35224 (31.9)	7004 (28.4)	11503 (28.4)	3629 (37.5)	10342 (36.8)	1659 (39.3)	1087 (32.8)
22.5–24.9	31582 (28.6)	7227 (29.3)	11969 (29.5)	2682 (27.7)	7727 (27.5)	1106 (26.2)	871 (26.3)
≥25	41652 (37.6)	10072 (40.6)	16496 (40.7)	3059 (31.6)	9409 (33.4)	1339 (31.8)	1277 (38.6)
Missing	82 (0.1)	24 (0.1)	32 (0.1)	8 (0.1)	13 (0.0)	4 (0.1)	1 (0.0)
**Smoking** (cigarettes/day)							
Non-smoker	24664 (22.3)	24664 (100)	0 (0)	0 (0)	0 (0)	0 (0)	0 (0)
Former smoker	40560 (36.7)	0 (0)	40560 (100)	0 (0)	0 (0)	0 (0)	0 (0)
1–9	10040 (9.1)	0 (0)	0 (0)	9668 (100)	0 (0)	372 (8.8)	0 (0)
10–20	23892 (21.6)	0 (0)	0 (0)	0 (0)	20046 (71.3)	3846 (91.2)	0 (0)
21–40	10587 (9.6)	0 (0)	0 (0)	0 (0)	7620 (27.1)	0 (0)	2967 (89.6)
≥40	812 (0.7)	0 (0)	0 (0)	0 (0)	467 (1.7)	0 (0)	345 (10.4)
**Alcohol drinking frequency** (times/week)							
Non-drinking	38642 (35.0)	11844 (48.0)	14710 (36.3)	2806 (29.0)	7290 (25.9)	1131 (26.8)	861 (26.0)
1–2	45915 (41.5)	8780 (35.6)	16864 (41.6)	4599 (47.6)	12604 (44.8)	1878 (44.5)	1190 (35.9)
3–4	14234 (12.9)	2070 (8.4)	4899 (12.1)	1229 (12.7)	4693 (16.7)	686 (16.3)	657 (19.8)
≥5	10715 (9.7)	1704 (6.9)	3616 (8.8)	947 (9.8)	3357 (11.9)	505 (12.0)	586 (17.8)
Missing	1049 (0.9)	266 (1.1)	471 (1.2)	87 (0.9)	189 (0.7)	18 (0.4)	18 (0.5)
**Physical exercise** (days/week)							
0–2	83939 (75.9)	18118 (73.5)	29378 (72.4)	7432 (76.9)	22798 (81.0)	3474 (82.4)	2739 (82.7)
≥3	24233 (21.9)	6020 (24.4)	10130 (25.0)	2052 (21.2)	4834 (17.2)	675 (16.0)	522 (15.8)
Missing	2383 (2.2)	526 (2.1)	1052 (2.6)	184 (1.9)	501 (1.8)	69 (1.6)	51 (1.5)
**Charlson comorbidity index**							
0	751 (0.7)	192 (0.8)	282 (0.7)	61 (0.6)	145 (0.5)	38 (0.9)	33 (1.0)
1	839 (0.8)	227 (0.9)	307 (0.8)	66 (0.7)	180 (0.6)	40 (0.9)	19 (0.6)
2	25494 (23.1)	5904 (23.9)	9140 (22.5)	2186 (22.6)	6473 (23.0)	1029 (24.4)	762 (23.0)
≥3	83471 (75.5)	18341 (74.4)	30831 (76.0)	7355 (76.1)	21335 (75.8)	3111 (73.8)	2498 (75.4)

a Four groups of the smoking trajectory model for pre-diagnosis current smokers (n=45331) in the entire study population.

Among the 110555 cancer patients, the five leading cancer types were stomach (28.2%), colon and rectum (19.1%), prostate (11.9%), liver (6.4%), and lung (6.1%) (Supplemental Table S4). During 918546 person-years of follow-up, 23888 deaths occurred. Lung cancer was the most common fatal cancer type (14.6%), followed by liver (12.3%), gastric (7.9%), colorectal (7.3%), and prostate (4.8%) cancers. Moreover, one-third of the deaths were due to causes other than cancer (n=8016; 33.6%).

### Mortality risks according to smoking trajectories

**Figure 2:**
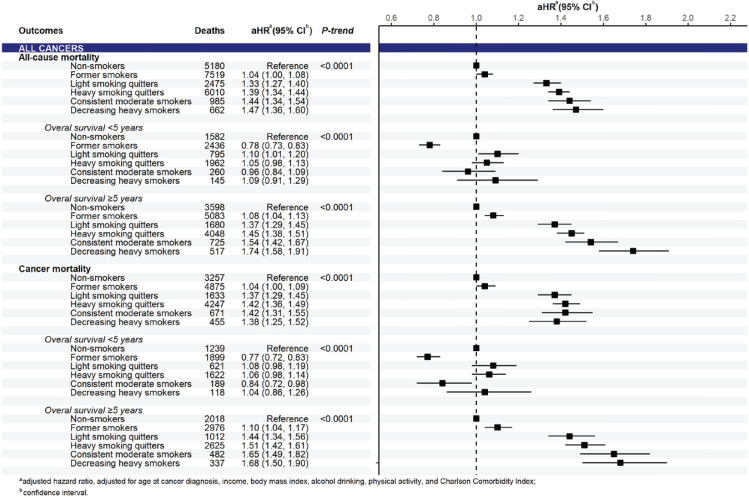
Mortality risk according to smoking trajectories for pooled cancers (n=110555)

**Figure 3:**
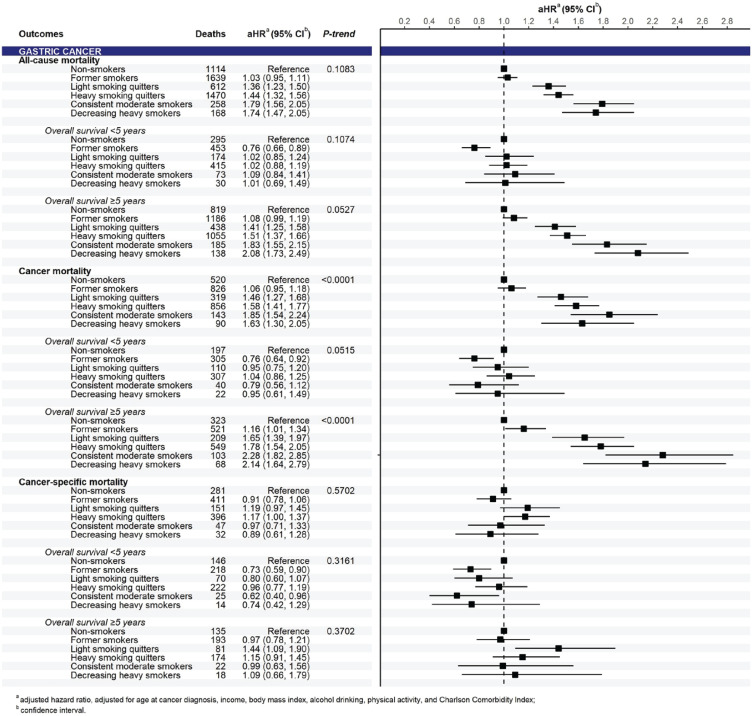
Mortality risk according to smoking trajectories for gastric cancer (n=31150)

**Figure 4:**
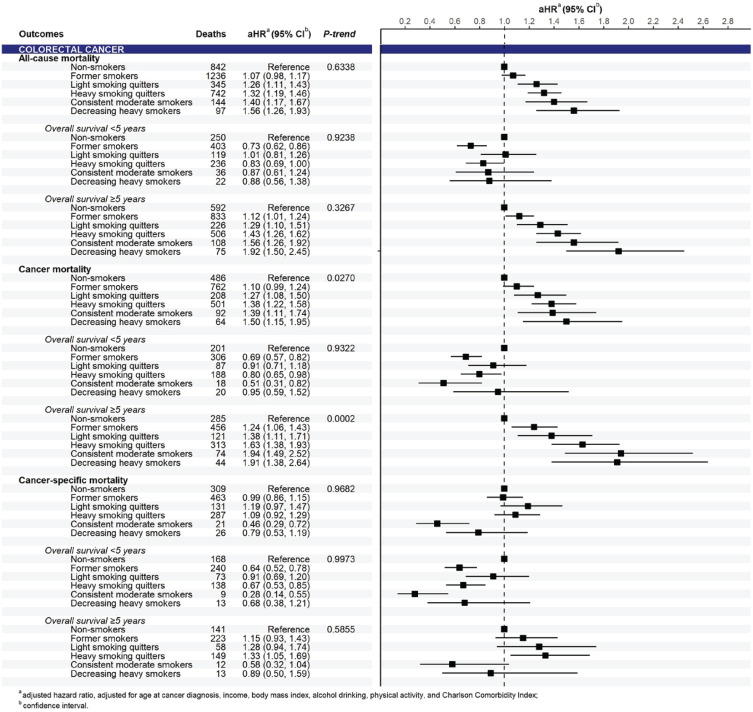
Mortality risk according to smoking trajectories for colorectal cancer (n=21069)

In the analysis of pooled cancers, compared to non-smokers, smoking significantly increased all-cause and cancer mortality risks, which were observed in all smoking trajectories of pre-diagnosis current smokers in a dose-response pattern (p<0.0001) (Figure 2). Significant associations between smoking trajectories and all-cause and cancer mortality were clearly present in the subgroup of 5-year cancer survivors only. In the analysis of cancer groups, we found a positive association between smoking trajectory and all-cause and cancer mortality risks for both pooled smoking-related cancers and pooled cancers unrelated to smoking in a dose-response manner in long-term survival (Supplementary file Figure S3). By cancer types, the smoking trajectory was associated with a significant increase in all-cause and cancer mortality risks in patients with gastric (Figure 3) and colorectal cancers (Figure 4) but not in those with liver and lung cancers (Supplementary file Figure S4). A significant association of smoking trajectory with cancer-specific mortality risk was found only in patients with lung cancer (Supplemental Figure S4). We also found a link between smoking and all-cause mortality risk in patients with other smoking-related cancer types, including cancers of the lip, oral cavity, pharynx, kidney, and bladder (Supplementary file Table S5), as well as in some cancer types that are unrelated to smoking, including prostate and thyroid cancers.

In the analysis of the three-measurement subpopulation, all-cause mortality risks for pooled cancers and gastric and colorectal cancers (Table 2) were the highest in heavy smokers, regardless of whether they quit after diagnosis (see Supplemental Table S6 for pooled cancer groups and liver and lung cancers). The results of the smoking status (Supplementary file Table S7) and pre-diagnosis smoking levels (Supplementary file Table S8) support our main findings; however, these approaches did not reveal a more important role of pre-diagnosis smoking levels in predicting cancer prognosis than that of post-diagnosis smoking patterns, as shown in the results of the smoking trajectory approach. The competing risks support our results in the primary analysis (Supplementary file Table S9).

**Table 2:** Mortality risk according to smoking trajectories for pooled cancers and gastric and colorectal cancers in the three-measurement subpopulation (N=43401)

*Smoking trajectories*	*Overall AHR (95% CI)*	*Overall survival <5 years AHR (95% CI)*	*Overall survival ≥5 years AHR (95% CI)*
**Pooled cancers**			
**All-cause mortality**			
Non-smokers (Ref.)			
Former smokers	1.12 (1.05–1.20)	0.93 (0.78–1.11)	1.15 (1.06–1.23)
Light smoking quitters	1.39 (1.26–1.53)	0.98 (0.76–1.27)	1.44 (1.30–1.59)
Heavy smoking quitters	1.51 (1.40–1.63)	1.11 (0.91–1.35)	1.54 (1.41–1.67)
Late heavy-smoking quitters	1.59 (1.39–1.82)	1.15 (0.77–1.73)	1.69 (1.47–1.95)
Heavy smoking relapse	1.67 (1.48–1.88)	0.86 (0.62–1.20)	1.75 (1.54–1.99)
Consistent heavy smokers	1.62 (1.43–1.84)	0.87 (0.61–1.23)	1.70 (1.48–1.95)
**Cancer mortality**			
Non-smokers (Ref.)			
Former smokers	1.15 (1.05–1.26)	0.84 (0.68–1.03)	1.21 (1.10–1.34)
Light smoking quitters	1.53 (1.35–1.72)	1.02 (0.77–1.36)	1.61 (1.41–1.83)
Heavy smoking quitters	1.59 (1.44–1.75)	1.09 (0.87–1.37)	1.64 (1.47–1.84)
Late heavy-smoking quitters	1.65 (1.40–1.95)	1.12 (0.70–1.77)	1.81 (1.51–2.17)
Heavy smoking relapse	1.67 (1.43–1.94)	0.68 (0.46–1.02)	1.86 (1.58–2.20)
Consistent heavy smokers	1.76 (1.50–2.05)	0.78 (0.52–1.17)	1.93 (1.63–2.28)
**Gastric cancer**			
**All-cause mortality**			
Non-smokers (Ref.)			
Former smokers	1.19 (1.03–1.37)	0.85 (0.57–1.27)	1.24 (1.06–1.45)
Light smoking quitters	1.54 (1.27–1.86)	1.10 (0.63–1.90)	1.60 (1.31–1.97)
Heavy smoking quitters	1.75 (1.49–2.05)	1.01 (0.67–1.54)	1.79 (1.51– 2.13)
Late heavy-smoking quitters	1.81 (1.36–2.41)	0.41 (0.14–1.21)	2.04 (1.52–2.76)
Heavy smoking relapse	1.96 (1.56–2.46)	0.61 (0.29–1.25)	2.15 (1.69–2.74)
Consistent heavy smokers	1.93 (1.47–2.52)	0.48 (0.18–1.27)	2.19 (1.65–2.90)
**Cancer mortality**			
Non-smokers (Ref.)			
Former smokers	1.25 (1.00–1.57)	0.82 (0.48–1.41)	1.36 (1.06–1.75)
Light smoking quitters	1.87 (1.41–2.49)	1.35 (0.67–2.72)	2.00 (1.46–2.74)
Heavy smoking quitters	2.19 (1.73–2.78)	1.32 (0.77–2.27)	2.28 (1.75–2.97)
Late heavy-smoking quitters	2.59 (1.77–3.80)	0.72 (0.23–2.29)	3.01 (1.99–4.54)
Heavy smoking relapse	2.26 (1.62–3.14)	0.31 (0.09–1.08)	2.80 (1.97–3.98)
Consistent heavy smokers	2.40 (1.66–3.48)	0.55 (0.16–1.91)	2.95 (1.99–4.37)
**Cancer-specific mortality**			
Non-smokers (Ref.)			
Former smokers	1.09 (0.78–1.52)	0.88 (0.46–1.67)	1.15 (0.77–1.71)
Light smoking quitters	1.42 (0.92–2.22)	0.91 (0.36–2.30)	1.59 (0.96–2.65)
Heavy smoking quitters	1.59 (1.11–2.26)	1.35 (0.71–2.56)	1.41 (0.91–2.19)
Late heavy-smoking quitters	0.56 (0.20–1.55)	0.57 (0.12–2.64)	0.42 (0.10–1.75)
Heavy smoking relapse	1.72 (1.04–2.86)	0.28 (0.06–1.28)	2.33 (1.34–4.06)
Consistent heavy smokers	0.73 (0.33–1.63)	0.47 (0.10–2.14)	0.78 (0.30–2.02)
**Colorectal cancer**			
**All-cause mortality**			
Non-smokers (Ref.)			
Former smokers	1.21 (1.01–1.44)	0.98 (0.59–1.62)	1.19 (0.98–1.45)
Light smoking quitters	1.45 (1.12–1.87)	0.72 (0.31–1.66)	1.58 (1.21–2.08)
Heavy smoking quitters	1.52 (1.22–1.89)	1.11 (0.62–1.98)	1.52 (1.20–1.93)
Late heavy-smoking quitters	1.35 (0.91–2.01)	2.58 (0.70–9.47)	1.46 (0.96–2.22)
Heavy smoking relapse	1.78 (1.30–2.43)	1.03 (0.37–2.86)	1.97 (1.42–2.74)
Consistent heavy smokers	2.00 (1.47–2.73)	1.19 (0.54–2.62)	1.97 (1.40–2.78)
**Cancer mortality**			
Non-smokers (Ref.)			
Former smokers	1.33 (1.04–1.70)	0.96 (0.54–1.71)	1.34 (1.02–1.76)
Light smoking quitters	1.63 (1.16–2.30)	0.98 (0.41–2.36)	1.79 (1.23–2.60)
Heavy smoking quitters	1.51 (1.13–2.02)	1.01 (0.51–1.98)	1.55 (1.11–2.15)
Late heavy-smoking quitters	1.42 (0.84–2.39)	2.18 (0.45–10.49)	1.61 (0.92–2.81)
Heavy smoking relapse	2.06 (1.38–3.06)	0.95 (0.30–3.02)	2.39 (1.56–3.67)
Consistent heavy smokers	2.53 (1.74–3.67)	1.12 (0.46–2.75)	2.63 (1.73–3.99)
**Cancer-specific mortality**			
Non-smokers (Ref.)			
Former smokers	1.13 (0.82–1.58)	1.06 (0.53–2.10)	1.05 (0.72–1.54)
Light smoking quitters	1.20 (0.74–1.95)	1.49 (0.58–3.80)	1.12 (0.63–1.99)
Heavy smoking quitters	1.24 (0.84–1.82)	1.03 (0.47–2.27)	1.17 (0.75–1.85)
Late heavy-smoking quitters	0.91 (0.41–2.01)	-	1.20 (0.53–2.69)
Heavy smoking relapse	0.78 (0.38–1.60)	0.90 (0.24–3.45)	0.72 (0.30–1.72)
Consistent heavy smokers	0.76 (0.37–1.56)	0.53 (0.15–1.83)	0.58 (0.22–1.48)

AHR: adjusted hazard ratio; adjusted for age at cancer diagnosis, income, body mass index, alcohol drinking, physical activity, and Charlson comorbidity index.

Compared to decreasing heavy smokers (T1.4), for pooled cancers, all-cause mortality risk was significantly lower (11–38%) for smoking trajectories among 5-year survivors (Supplementary file Table S10). A risk reduction in heavy smoking quitters (T1.2) for specific cancer types was observed for gastric and colorectal cancers in long-term survival. Former smokers who quit at least 6 years before cancer diagnosis had a consistently lower risk of all-cause mortality in the pooled analysis of all cancers and gastric and colorectal cancers.

### Sensitivity analysis

Mortality risks according to smoking trajectories resulted from analyses with time-dependent covariates that were consistent with the results of the main analyses (Supplementary file Tables S11 and S12). The trajectory analysis performed for the subgroups of multiple post-diagnosis measurements showed that more than 75% of the patients retained their post-diagnosis smoking behavior (Supplementary file Table S13 and Figure S5).

## DISCUSSION

This study was conducted among Korean male cancer survivors, eliciting several findings. Current smokers accounted for 41% of participants at pre-diagnosis and 31% continued to smoke, comparable to that reported in a study of Korean men with colorectal cancer^4^. Supporting our results on elevated all-cause mortality risk in all trajectories of pre-diagnosis current smokers with colorectal cancer, specifically, they found a significant increase of 21–30% in all-cause mortality risk associated with pre-diagnosis smoking, regardless of post-diagnosis smoking status^4^. In particular, they observed the association of smoking with all-cause mortality restricted to the surgical treatment group^4^, also reported in another study^17^. Explanations were suggested, possibly relating to surgery complications^17^ or lower baseline hazards in the surgical group than in other treatment groups^4^. A qualitative synthesis indicated that continued smoking might contribute to an elevated incidence of late, but not acute, toxicities^8^. In our study, the toxicities of smoking in long-term survival and their associations with mortality appeared to be more evident in less fatal cancer types, i.e. in gastric and colorectal cancers (5-year relative survival rates (2014–2018) among Korean men, 77.8% and 75.6%, respectively), but not in lung and liver cancers (27.0% and 37.8%, respectively)^31^. The findings were shown for all-cause and cancer mortality and were supported by competing risk analysis. In particular, significant associations between smoking and mortality risks were also found in the group of cancers unrelated to smoking. Therefore, we suggest that tobacco smoking is an independent risk factor for cancer prognosis among male cancer patients because of its impact on general health and cancer progression. The impact of smoking on mortality risk was not observed in cancer patients who survived <5 years or were diagnosed with fatal cancers, such as liver or lung cancers, which could be explained by the fact that their prognosis might be predominantly affected by other clinical factors such as cancer phase and treatments rather than by behavioral risk factors. In the case of lung cancer, significant associations were observed with cancer-specific death. This might be because lung cancer patients with poor prognosis may be at a higher risk of death from lung cancer itself rather than from other causes. Up to 44.8% of Korean men with lung cancer were diagnosed at advanced stages, with an estimated 5-year relative survival rates of 7.0% only^31^. More biological evidence on the impact of smoking on cancer progression is necessary; however, this is not limited to smoking-related cancers.

Referring to decreasing heavy smokers, heavy smoking quitters were at a significantly lower risk of death from any cause, suggesting the positive effects of complete smoking cessation. Therefore, we strongly recommend that cancer patients to quit smoking as soon as possible after diagnosis. It is never too late for smoking cessation, even for those who smoked heavily before the cancer diagnosis. Smoking cessation services should be strengthened in cancer survivorship care. Proactive support should be provided to heavy smokers to help them quit entirely after cancer diagnosis, as some patients tend to relapse to smoking post-diagnosis. Determinants of smoking continuation among cancer survivors should be further investigated to aid in this.

Using trajectory analysis, this study revealed a more comprehensive profile of smoking patterns among cancer survivors when smoking status and amount were considered simultaneously. A study on smoking trajectories identified smoking patterns different from ours, including low steady, rise and fall, lowering, rise and sharp fall, high steady, and very high steady^27^. This discrepancy might be due to the differences in the study population and objectives. While their study tracked the smoking trajectory over a long period in terms of development patterns among young adults, we investigated a cancer population in which cancer diagnosis possibly played a predominant role in affecting behavior change. In particular, we identified groups of pre-diagnosis heavy smokers who delayed quitting or relapsed into smoking after quitting post-diagnosis. The participants in these groups were at a high risk of death, comparable to that of consistent heavy smokers. Those who tended to retain their pre-diagnosis smoking behavior were mostly heavy smokers, suggesting that intensive and continuous support is needed for patients who smoke heavily. In addition, the dose-response association of smoking trajectories with all-cause and cancer mortality risks was shown in the pooled analysis of all cancers and smoking-related cancer types, with the pre-diagnosis smoking levels possibly predicting patient prognosis better than the post-diagnosis smoking status, which was not revealed in the conventional approaches based on smoking status and pre-diagnosis smoking classification.

### Strengths and limitations

This study has several strengths. First, this was a population-based cohort study with a large sample size. A large number of patients with cancer enabled us to analyze the pooled sample of all cancers and some specific cancer types. Second, we used trajectory analysis to comprehensively investigate smoking patterns among cancer survivors, while previous studies on this population were mainly based on smoking status^4-6,8,14,16,17^. In addition, we were able to perform a competing risk analysis given that a large proportion of cancer survivors died from causes other than cancer. The results of the competing risk analysis supported our findings in the primary analysis. Our study also has limitations. First, we could not include people who did not undergo the national health examination post-diagnosis and excluded female patients and those with missing data on smoking behavior within appropriate time, which may lead to selection bias and limit the generalizability of our findings to the cancer male population. Second, this study used claims data that were not collected primarily for research purposes. To provide more conservative results related to the primary cancer diagnosis, we used the code ‘V193’ to confirm cancer cases ascertained using the ICD-code. A recent study validated the utility of primary diagnosis in the NHIS database in cancer research with high sensitivity (>90%) and accuracy (80% consistency with cancer registry data)^32^. Third, we examined smoking patterns with a limited number of repeated measurements, calling for further longitudinal studies tracking for more time points on patients’ behaviors during survivorship. The imputation of smoking level for the third measurement in the trajectory analysis was a trade-off for the study sample size (n=110555 with imputation vs 43401 without imputation). Nevertheless, the results derived from the three-measurement subpopulation supported our main findings. Furthermore, there might be residual confounding that we could not adjust for. We lacked clinical information, including cancer stage, pathology, and metastasis, which might greatly impact patients’ prognosis. A meta-analysis of cohort studies did not find changes in the estimates of the association between smoking status or cessation and overall or colorectal-specific survival with further adjustment for cancer stage^6^. Nonetheless, this limitation reduces the ability to make inferences about the mortality risk according to smoking trajectories in our study. The reasons for quitting smoking after a cancer diagnosis might also be confounding. Those who quit for risk reduction while being relatively healthy and those who stop smoking because of poor health might have different prognosis^33^. Due to data unavailability, this factor was not accounted for in our analysis; this might affect our association estimates. Despite these limitations, we provided robust evidence of the health benefits of smoking cessation among male cancer patients in long-term survival, supported by a recent study on tobacco use-related cancers. They reported protective effects observed over the 5 years following smoking cessation among Non-Hispanic Black patients, marked by the reduced risk of a high neutrophil-to-lymphocyte ratio (i.e. an inflammation biomarker used in cancer prevention and management programs to indicate the influence of smoking cessation)^34^.

## CONCLUSIONS

Smoking significantly increased all-cause and cancer mortality risks among male cancer patients in pooled all cancers, pooled smoking-related cancers, and pooled cancers unrelated to smoking. Smoking also increased all-cause and cancer mortality risks in patients with gastric and colorectal cancers and cancer-specific mortality risks in patients with lung cancer. The significant associations between smoking and mortality risks were presented more clearly among 5-year survivors and those with less fatal cancer types. Smoking cessation post-cancer diagnosis is beneficial for male cancer patients, even among those who smoke heavily.

## Data Availability

The data used in this study (NHIS-2020-1-237) were provided by the National Health Insurance Service. They are available for researchers who meet the criteria for access to confidential data. For the protection of personal information, the data cannot be shared because NHIS prohibits the transfer, rental, or sale of the database to third parties except for researchers who have been approved for access. The NHIS data can be requested through its website (https://nhiss.nhis.or.kr).
