# Supplementary material for: Mortality risk according to smoking trajectories after cancer diagnosis among Korean male cancer survivors: A population-based cohort study
Source: Tob Induc Dis. 2023 May 25;21:69. doi: 10.18332/tid/163175 (PMC10210093; doi:10.18332/tid/163175)
Supplement: Supplementary file 1 [file TID-21-69-s1.pdf]

SUPPLEMENTAL MATERIAL

Supplemental Figure S1. Flowchart of the cohort study

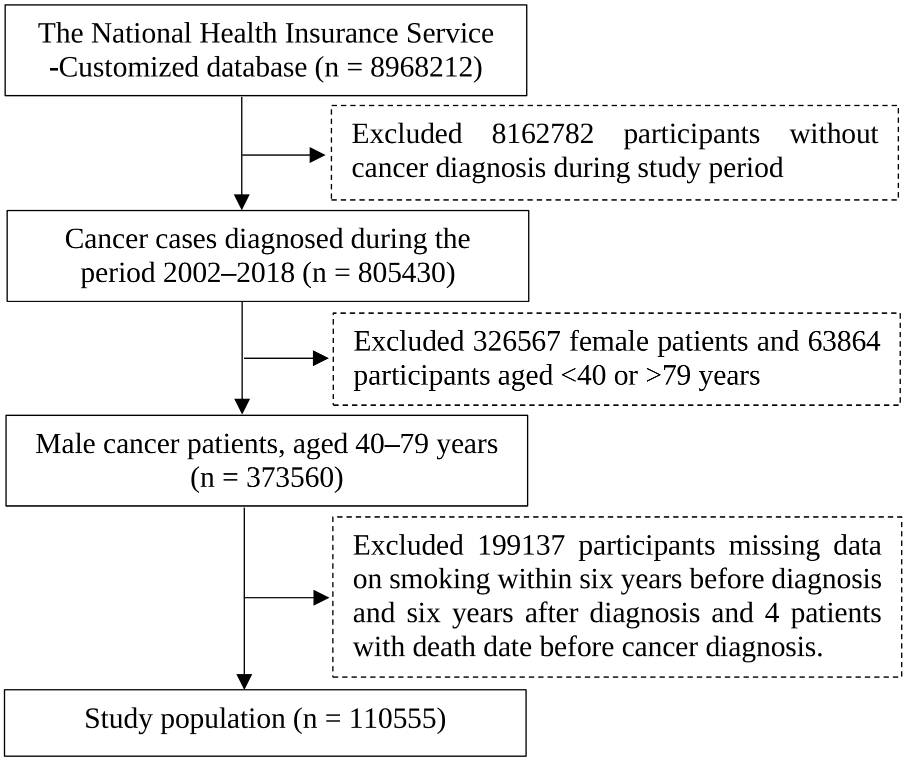

Supplemental Figure S2. Survival curves for (A) smoking trajectories for the entire study population, (B) smoking trajectories for the three-measurement subpopulation, (C) smoking status, and (D) baseline smoking levels

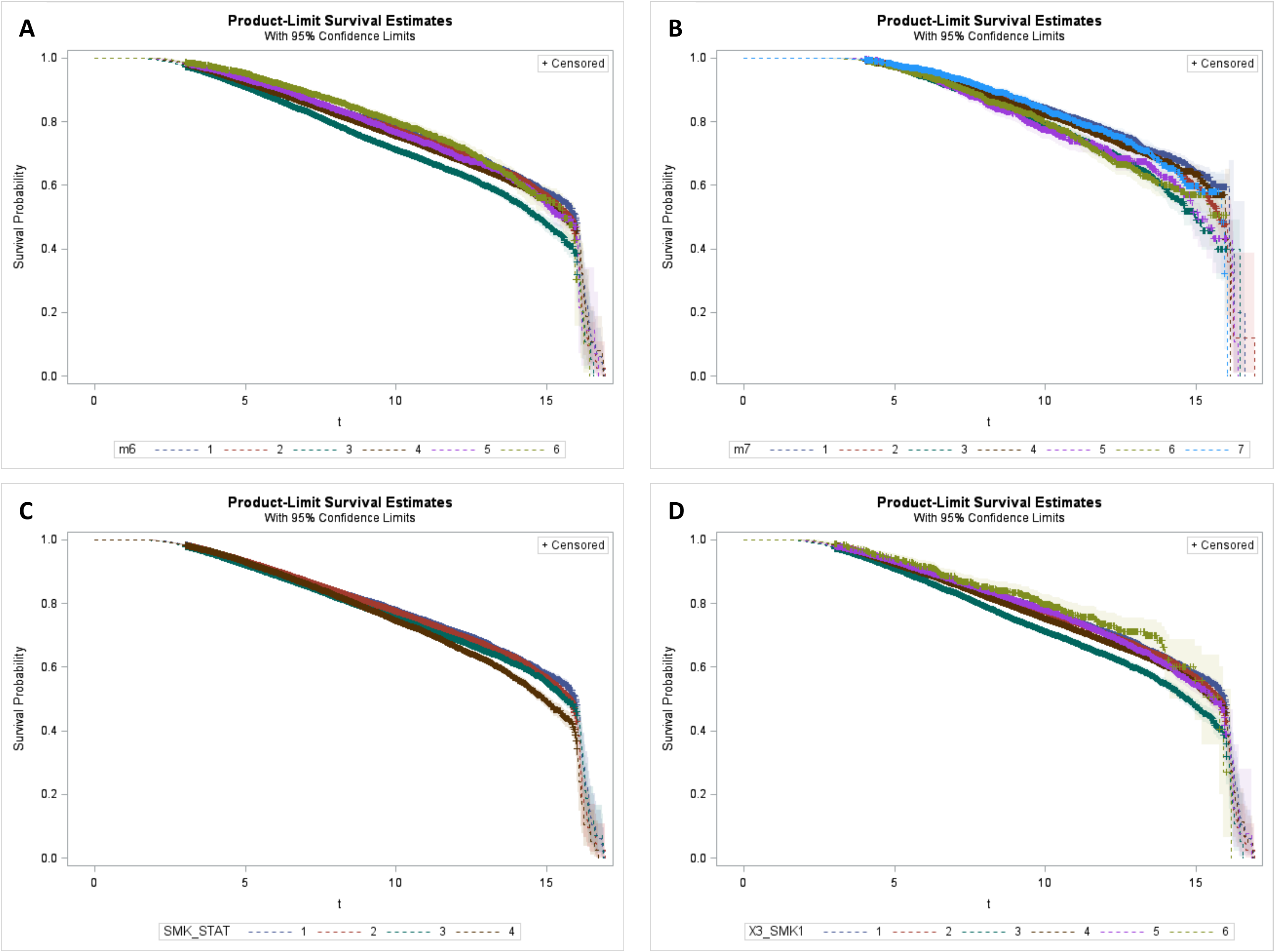

Supplemental Figure S3. Mortality risks according to smoking trajectories for pooled cancer groups: smoking-related cancers and cancers unrelated to smoking

Outcomes	Deaths	aHR (95% CI)	*P-trend*
**SMOKING-RELATED CANCERS**			
**All-cause mortality**			
Non-smokers	3667	Reference	0.0002
Former smokers	5609	1.03 (0.99, 1.07)	
Light smoking quitters	1934	1.31 (1.24, 1.38)	
Heavy smoking quitters	4940	1.35 (1.30, 1.42)	
Consistent moderate smokers	777	1.40 (1.29, 1.51)	
Decreasing heavy smokers	534	1.45 (1.32, 1.59)	
Overall survival <5 years			
Non-smokers	1138	Reference	0.0134
Former smokers	1818	0.76 (0.70, 0.82)	
Light smoking quitters	631	1.03 (0.93, 1.14)	
Heavy smoking quitters	1630	0.98 (0.91, 1.06)	
Consistent moderate smokers	207	0.89 (0.77, 1.04)	
Decreasing heavy smokers	114	1.02 (0.84, 1.24)	
Overall survival ≥5 years			
Non-smokers	2529	Reference	<0.0001
Former smokers	3791	1.08 (1.03, 1.14)	
Light smoking quitters	1303	1.36 (1.27, 1.45)	
Heavy smoking quitters	3310	1.43 (1.35, 1.51)	
Consistent moderate smokers	570	1.53 (1.39, 1.68)	
Decreasing heavy smokers	420	1.76 (1.58, 1.95)	
**Cancer mortality**			
Non-smokers	2321	Reference	<0.0001
Former smokers	3644	1.02 (0.97, 1.08)	
Light smoking quitters	1270	1.33 (1.24, 1.42)	
Heavy smoking quitters	3493	1.38 (1.30, 1.45)	
Consistent moderate smokers	530	1.38 (1.25, 1.52)	
Decreasing heavy smokers	360	1.33 (1.18, 1.48)	
Overall survival <5 years			
Non-smokers	917	Reference	0.0076
Former smokers	1441	0.74 (0.68, 0.81)	
Light smoking quitters	488	0.98 (0.88, 1.10)	
Heavy smoking quitters	1352	0.97 (0.89, 1.06)	
Consistent moderate smokers	146	0.74 (0.62, 0.88)	
Decreasing heavy smokers	94	0.98 (0.79, 1.21)	
Overall survival ≥5 years			
Non-smokers	1404	Reference	<0.0001
Former smokers	2203	1.10 (1.03, 1.18)	
Light smoking quitters	782	1.43 (1.31, 1.57)	
Heavy smoking quitters	2141	1.50 (1.40, 1.61)	
Consistent moderate smokers	384	1.67 (1.49, 1.87)	
Decreasing heavy smokers	266	1.67 (1.46, 1.91)	
**CANCERS UNRELATED TO SMOKING**			
**All-cause mortality**			
Non-smokers	1513	Reference	0.0651
Former smokers	1910	1.05 (0.98, 1.12)	
Light smoking quitters	541	1.36 (1.24, 1.51)	
Heavy smoking quitters	1070	1.43 (1.32, 1.55)	
Consistent moderate smokers	208	1.52 (1.31, 1.76)	
Decreasing heavy smokers	128	1.50 (1.24, 1.80)	
Overall survival <5 years			
Non-smokers	444	Reference	0.0386
Former smokers	618	0.81 (0.72, 0.92)	
Light smoking quitters	164	1.22 (1.02, 1.46)	
Heavy smoking quitters	332	1.12 (0.97, 1.30)	
Consistent moderate smokers	53	1.10 (0.82, 1.47)	
Decreasing heavy smokers	31	1.25 (0.86, 1.80)	
Overall survival ≥5 years			
Non-smokers	1069	Reference	0.0776
Former smokers	1292	1.07 (0.99, 1.16)	
Light smoking quitters	377	1.40 (1.25, 1.58)	
Heavy smoking quitters	738	1.47 (1.33, 1.62)	
Consistent moderate smokers	155	1.58 (1.33, 1.87)	
Decreasing heavy smokers	97	1.65 (1.33, 2.04)	
**Cancer mortality**			
Non-smokers	936	Reference	<0.0001
Former smokers	1231	1.07 (0.98, 1.16)	
Light smoking quitters	363	1.44 (1.28, 1.63)	
Heavy smoking quitters	754	1.49 (1.35, 1.65)	
Consistent moderate smokers	141	1.51 (1.26, 1.81)	
Decreasing heavy smokers	95	1.50 (1.21, 1.86)	
Overall survival <5 years			
Non-smokers	322	Reference	0.0009
Former smokers	458	0.82 (0.71, 0.94)	
Light smoking quitters	133	1.34 (1.09, 1.64)	
Heavy smoking quitters	270	1.19 (1.01, 1.41)	
Consistent moderate smokers	43	1.13 (0.82, 1.56)	
Decreasing heavy smokers	24	1.20 (0.79, 1.83)	
Overall survival ≥5 years			
Non-smokers	614	Reference	0.0001
Former smokers	773	1.10 (0.99, 1.22)	
Light smoking quitters	230	1.46 (1.25, 1.70)	
Heavy smoking quitters	484	1.53 (1.35, 1.73)	
Consistent moderate smokers	98	1.55 (1.25, 1.93)	
Decreasing heavy smokers	71	1.71 (1.33, 2.20)	

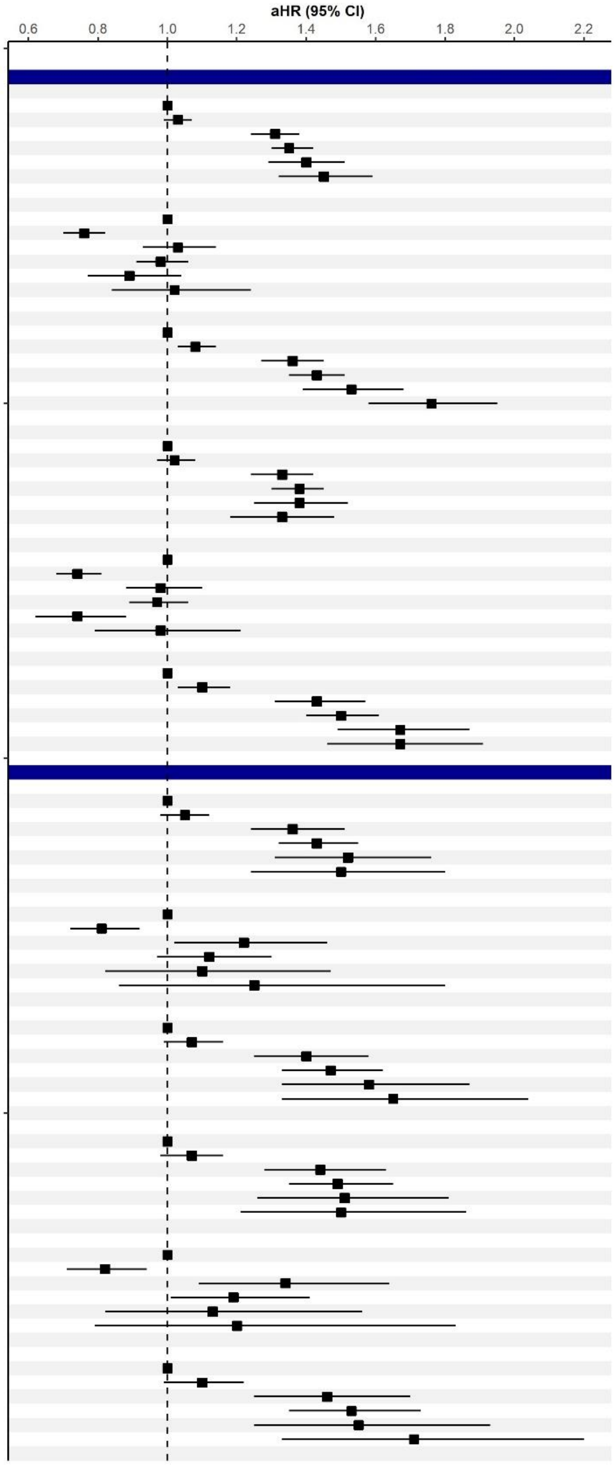

aHR: adjusted hazard ratio, adjusted for age at cancer diagnosis, income, body mass index, alcohol drinking, physical activity, and Charlson Comorbidity Index; CI: confidence interval.

Supplemental Figure S4. Mortality risks according to smoking trajectories for liver cancer (n=7068) and lung cancer (n=6707)

Outcomes	Deaths	aHR (95% CI)	*P-trend*
**LIVER CANCER**			
**All-cause mortality**			
Non-smokers	629	Reference	0.4787
Former smokers	875	1.06 (0.96, 1.18)	
Light smoking quitters	304	1.20 (1.04, 1.38)	
Heavy smoking quitters	736	1.17 (1.05, 1.31)	
Consistent moderate smokers	122	0.99 (0.81, 1.21)	
Decreasing heavy smokers	96	1.22 (0.97, 1.52)	
Overal survival <5 years			
Non-smokers	247	Reference	0.3936
Former smokers	342	0.78 (0.66, 0.92)	
Light smoking quitters	123	0.98 (0.79, 1.22)	
Heavy smoking quitters	303	0.97 (0.81, 1.15)	
Consistent moderate smokers	39	0.84 (0.60, 1.18)	
Decreasing heavy smokers	22	0.96 (0.62, 1.50)	
Overal survival ≥5 years			
Non-smokers	382	Reference	0.2938
Former smokers	533	1.15 (1.01, 1.32)	
Light smoking quitters	181	1.20 (1.01, 1.44)	
Heavy smoking quitters	433	1.19 (1.03, 1.37)	
Consistent moderate smokers	83	1.06 (0.83, 1.35)	
Decreasing heavy smokers	74	1.48 (1.14, 1.91)	
**Cancer mortality**			
Non-smokers	549	Reference	0.6069
Former smokers	761	1.04 (0.93, 1.17)	
Light smoking quitters	257	1.15 (0.99, 1.34)	
Heavy smoking quitters	639	1.13 (1.00, 1.28)	
Consistent moderate smokers	106	0.98 (0.79, 1.21)	
Decreasing heavy smokers	78	1.09 (0.86, 1.40)	
Overal survival <5 years			
Non-smokers	227	Reference	0.4409
Former smokers	319	0.80 (0.67, 0.95)	
Light smoking quitters	109	0.95 (0.75, 1.20)	
Heavy smoking quitters	279	0.96 (0.80, 1.15)	
Consistent moderate smokers	37	0.86 (0.60, 1.22)	
Decreasing heavy smokers	19	0.89 (0.55, 1.43)	
Overal survival ≥5 years			
Non-smokers	322	Reference	0.4196
Former smokers	442	1.11 (0.96, 1.29)	
Light smoking quitters	148	1.16 (0.95, 1.41)	
Heavy smoking quitters	360	1.13 (0.96, 1.32)	
Consistent moderate smokers	69	1.03 (0.79, 1.34)	
Decreasing heavy smokers	59	1.34 (1.01, 1.79)	
**Cancer-specific mortality**			
Non-smokers	470	Reference	0.8389
Former smokers	658	1.04 (0.92, 1.17)	
Light smoking quitters	213	1.10 (0.94, 1.30)	
Heavy smoking quitters	547	1.10 (0.96, 1.25)	
Consistent moderate smokers	87	0.90 (0.71, 1.14)	
Decreasing heavy smokers	55	0.87 (0.66, 1.16)	
Overal survival <5 years			
Non-smokers	199	Reference	0.4390
Former smokers	280	0.79 (0.66, 0.95)	
Light smoking quitters	95	0.92 (0.72, 1.18)	
Heavy smoking quitters	250	0.95 (0.79, 1.16)	
Consistent moderate smokers	32	0.82 (0.56, 1.19)	
Decreasing heavy smokers	16	0.83 (0.50, 1.39)	
Overal survival ≥5 years			
Non-smokers	271	Reference	0.7663
Former smokers	378	1.11 (0.95, 1.31)	
Light smoking quitters	118	1.09 (0.88, 1.36)	
Heavy smoking quitters	297	1.06 (0.90, 1.27)	
Consistent moderate smokers	55	0.94 (0.70, 1.26)	
Decreasing heavy smokers	39	1.02 (0.72, 1.44)	
**LUNG CANCER**			
**All-cause mortality**			
Non-smokers	346	Reference	0.6338
Former smokers	733	1.01 (0.89, 1.15)	
Light smoking quitters	249	1.19 (1.01, 1.40)	
Heavy smoking quitters	974	1.16 (1.02, 1.31)	
Consistent moderate smokers	76	1.20 (0.93, 1.55)	
Decreasing heavy smokers	54	1.22 (0.91, 1.63)	
Overal survival <5 years			
Non-smokers	147	Reference	0.9238
Former smokers	313	0.89 (0.73, 1.09)	
Light smoking quitters	102	1.15 (0.89, 1.49)	
Heavy smoking quitters	403	1.07 (0.88, 1.30)	
Consistent moderate smokers	27	0.97 (0.64, 1.48)	
Decreasing heavy smokers	12	0.94 (0.52, 1.71)	
Overal survival ≥5 years			
Non-smokers	199	Reference	0.3267
Former smokers	420	1.09 (0.92, 1.29)	
Light smoking quitters	147	1.30 (1.05, 1.62)	
Heavy smoking quitters	571	1.27 (1.07, 1.49)	
Consistent moderate smokers	49	1.32 (0.96, 1.81)	
Decreasing heavy smokers	42	1.69 (1.20, 2.37)	
**Cancer mortality**			
Non-smokers	282	Reference	0.1692
Former smokers	587	0.97 (0.84, 1.12)	
Light smoking quitters	192	1.11 (0.92, 1.33)	
Heavy smoking quitters	776	1.07 (0.93, 1.23)	
Consistent moderate smokers	62	1.18 (0.90, 1.56)	
Decreasing heavy smokers	44	1.13 (0.82, 1.56)	
Overal survival <5 years			
Non-smokers	136	Reference	0.0565
Former smokers	278	0.85 (0.69, 1.05)	
Light smoking quitters	92	1.13 (0.86, 1.47)	
Heavy smoking quitters	360	1.02 (0.83, 1.25)	
Consistent moderate smokers	24	0.94 (0.60, 1.47)	
Decreasing heavy smokers	10	0.85 (0.44, 1.62)	
Overal survival ≥5 years			
Non-smokers	146	Reference	0.0193
Former smokers	309	1.07 (0.87, 1.30)	
Light smoking quitters	100	1.18 (0.91, 1.52)	
Heavy smoking quitters	416	1.17 (0.96, 1.42)	
Consistent moderate smokers	38	1.34 (0.93, 1.93)	
Decreasing heavy smokers	34	1.65 (1.13, 2.41)	
**Cancer-specific mortality**			
Non-smokers	207	Reference	0.0982
Former smokers	476	1.07 (0.91, 1.26)	
Light smoking quitters	163	1.28 (1.04, 1.58)	
Heavy smoking quitters	645	1.20 (1.02, 1.41)	
Consistent moderate smokers	44	1.19 (0.85, 1.65)	
Decreasing heavy smokers	28	0.98 (0.66, 1.46)	
Overal survival <5 years			
Non-smokers	121	Reference	0.0472
Former smokers	249	0.87 (0.70, 1.08)	
Light smoking quitters	85	1.19 (0.89, 1.57)	
Heavy smoking quitters	325	1.04 (0.84, 1.29)	
Consistent moderate smokers	20	0.90 (0.56, 1.47)	
Decreasing heavy smokers	9	0.85 (0.43, 1.68)	
Overal survival ≥5 years			
Non-smokers	86	Reference	0.0066
Former smokers	227	1.33 (1.04, 1.71)	
Light smoking quitters	78	1.57 (1.15, 2.14)	
Heavy smoking quitters	320	1.52 (1.19, 1.93)	
Consistent moderate smokers	24	1.51 (0.96, 2.40)	
Decreasing heavy smokers	19	1.55 (0.94, 2.56)	

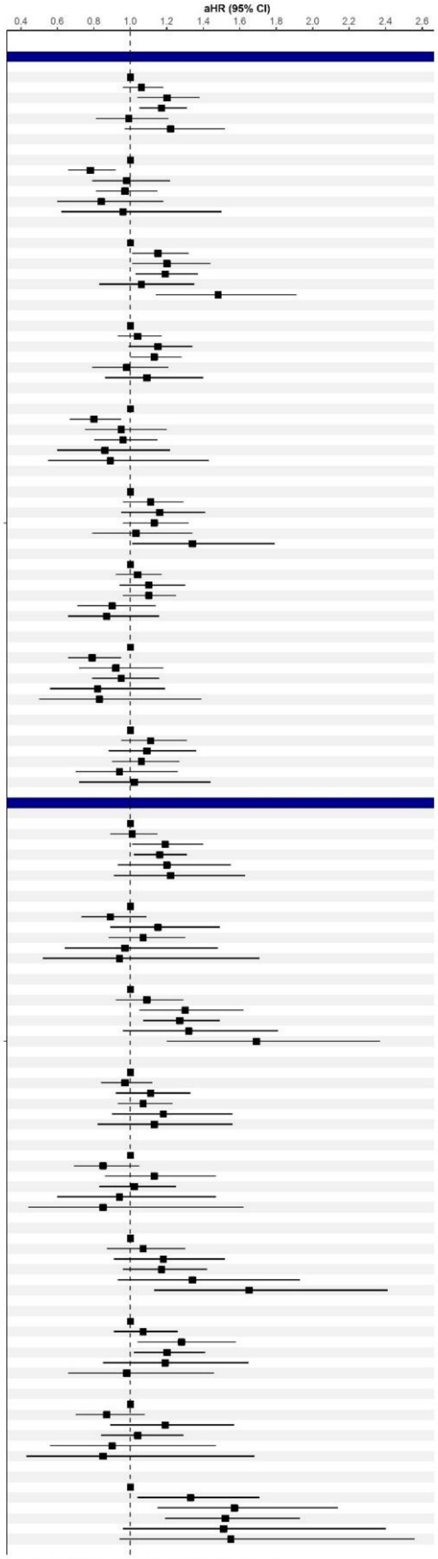

aHR: adjusted hazard ratio, adjusted for age at cancer diagnosis, income, body mass index, alcohol drinking, physical activity, and Charlson Comorbidity Index; CI: confidence interval.

Supplemental Figure S5. Smoking trajectories during post-diagnosis for the subgroups of (A) three post-diagnosis measurements (n=24340), (B) four post-diagnosis measurements (n=16257), (C) five post-diagnosis measurements (n=10012), and (D) six post-diagnosis measurements (n=5871)

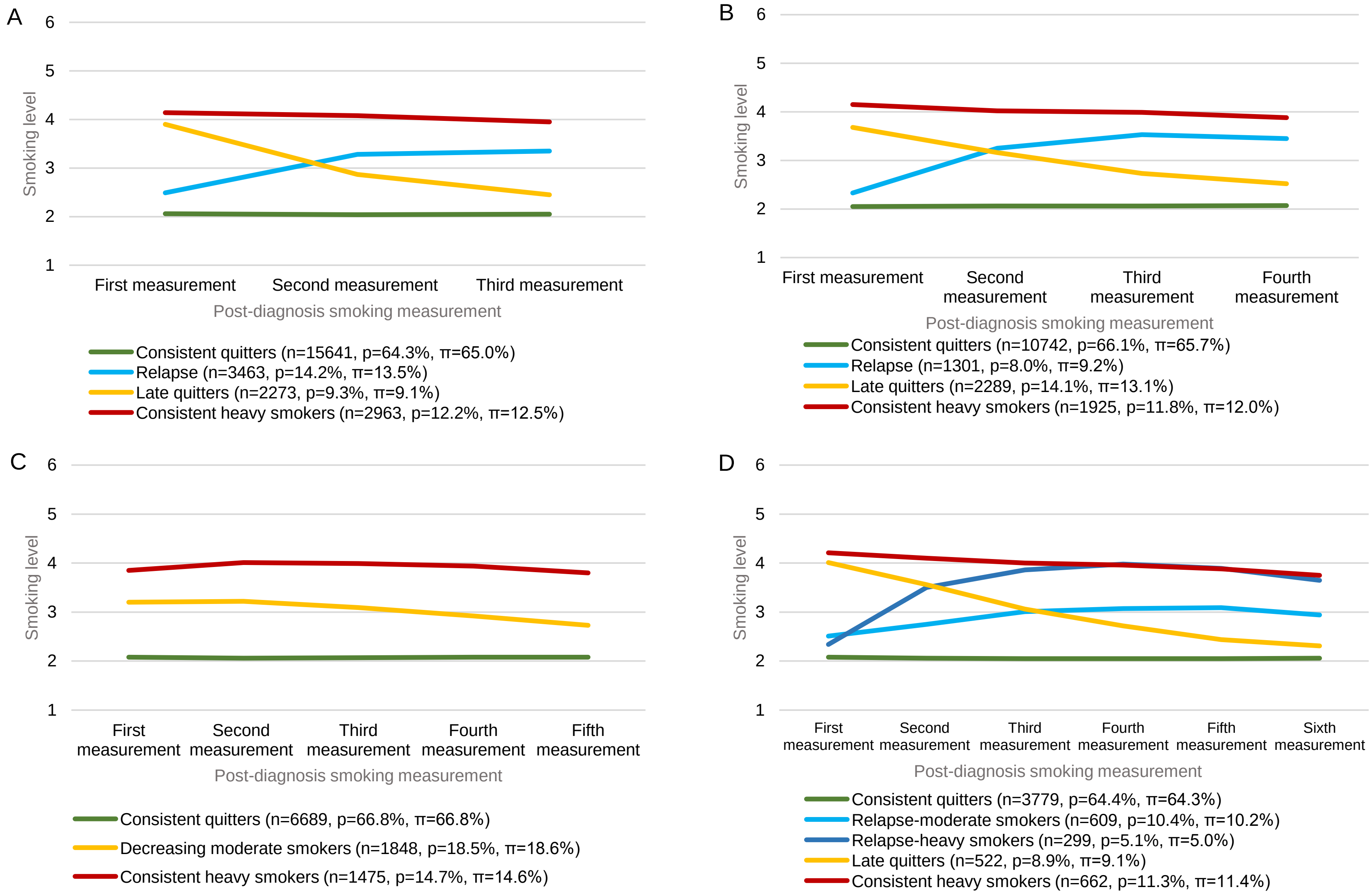

π: estimated group membership of the smoking trajectory (%); n: number of participants; p: percentage of participants assigned to the smoking trajectory group (%).

Supplemental Table S1. Model selection for smoking trajectories of pre-diagnosis current smokers during cancer diagnosis

Number of groups	BIC[a] (order = 2)	Selected (Yes/No)	Best fit model	BIC
**Pre-diagnosis current smokers in the study population** (n[b]=45331)-Model A (2,2,2,2)				
1	-160795	No		
2	-122919	No		
3	-115281	No		
4	-111743	Yes	2222	-111743
5	-111742	No		
6	-86337	No		
7	-80684	No		
**Pre-diagnosis current smokers in the three-measurement subpopulation** (n=18043)-Model B (2,2,2,2,2)				
1	-64213	No		
2	-56245	No		
3	-55020	No		
4	-53306	No		
5	-49746	Yes	22222	-49746
6	-48186	No		
7	-47642	No		

[a] Bayesian Information Criterion;
[b] number of participants.

Supplemental Table S2. Model details and evaluation for smoking trajectories of pre-diagnosis current smokers during cancer diagnosis

Group	n[a]	p[b]	π[c]	AvePP[d]	OCC[e]
**Pre-diagnosis current smokers in the study population** (n=45331)-Model A (2,2,2,2)					
Light smoking quitters	9668	21.3	22.3	0.99	278.6
Heavy smoking quitters	28133	62.1	60.9	0.98	27.3
Consistent moderate smokers	4218	9.3	9.6	0.98	557.7
Decreasing heavy smokers	3312	7.3	7.2	0.96	301.4
**Pre-diagnosis current smokers in the three-measurement subpopulation** (n=18043)-Model B (2,2,2,2,2)					
Light smoking quitters	3503	19.4	20.2	0.98	199.5
Heavy smoking quitters	9512	52.7	50.8	0.95	19.9
Late heavy smoking quitters	1272	7.0	6.9	0.95	259.3
Heavy smoking relapse	1890	10.5	11.2	0.94	124.6
Consistent heavy smokers	1866	10.3	10.8	1.00	1802.5

[a] number of participants;
[b] percentage of participants assigned to the smoking trajectory group (%);
[c] estimated group membership of the smoking trajectory (%);
[d] average posterior probability;
[e] odds of correct classification.

Supplemental Table S3. General characteristics of the three-measurement subpopulation at the study entry (2002-2003) (n=43401)

	**Three-measurement subpopulation** (n[a]=43401)	**Non-smokers** (n=8440)	**Former smokers** (n=16918)	**(T2.1) Light smoking quitters**[b] (n=3503)	**(T2.2) Heavy smoking quitters**[b] (n=9512)	**(T2.3) Late heavy smoking quitters**[b] (n=1272)	**(T2.4) Heavy smoking relapse**[b] (n=1890)	**(T2.5) Consistent heavy smokers**[b] (n=1866)
**Age,** mean (SD[c])	54.5 (8.9)	56.2 (9)	55.6 (8.9)	55.5 (9.2)	52.1 (8.2)	53.4 (8.3)	52.7 (8.5)	50.7 (7.5)
**Age group,** n (%)								
40–49	14538 (33.5)	2259 (26.8)	4863 (28.7)	1085 (31)	4152 (43.7)	453 (35.6)	794 (42)	932 (49.3)
50–59	15234 (35.1)	2901 (34.4)	5994 (35.4)	1089 (31.1)	3433 (36.1)	516 (40.6)	633 (33.5)	668 (35.3)
60–69	11620 (26.8)	2752 (32.6)	5091 (30.1)	1124 (32.1)	1740 (18.3)	265 (20.8)	402 (21.3)	246 (13)
70–79	2009 (4.6)	528 (6.3)	970 (5.7)	205 (5.9)	187 (2)	38 (3)	61 (3.2)	20 (1.1)
**Age at cancer diagnosis,** mean (SD)	61.7 (8.8)	63.1 (8.9)	63.1 (8.8)	62.5 (9.1)	59.3 (8.2)	60 (8.1)	59.8 (8.4)	57.4 (7.4)
**Income**, n (%)								
1st quintile	4264 (9.8)	765 (9.1)	1602 (9.5)	366 (10.4)	904 (9.5)	153 (12)	241 (12.8)	233 (12.3)
2nd quintile	4743 (10.9)	872 (10.3)	1773 (10.5)	410 (11.7)	1069 (11.2)	158 (12.4)	234 (12.4)	227 (12)
3rd quintile	7970 (18.4)	1425 (16.9)	3010 (17.8)	628 (17.9)	1800 (18.9)	305 (24)	418 (22.1)	384 (20.3)
4th quintile	9240 (21.3)	1757 (20.8)	3525 (20.8)	736 (21)	2127 (22.4)	266 (20.9)	419 (22.2)	410 (21.7)
5th quintile	16127 (37.2)	3444 (40.8)	6615 (39.1)	1275 (36.4)	3338 (35.1)	356 (28)	530 (28)	569 (30.1)
Missing	1057 (2.4)	177 (2.1)	393 (2.3)	88 (2.5)	274 (2.9)	34 (2.7)	48 (2.5)	43 (2.3)
**Body mass index** (kg/m^2), n (%)								
<18.5	719 (1.7)	103 (1.2)	203 (1.2)	86 (2.5)	202 (2.1)	27 (2.1)	58 (3.1)	40 (2.1)
18.5–22.4	13619 (31.4)	2360 (28)	4780 (28.3)	1266 (36.1)	3358 (35.3)	481 (37.8)	704 (37.2)	670 (35.4)
22.5–24.9	12563 (28.9)	2540 (30.1)	5017 (29.7)	989 (28.2)	2680 (28.2)	349 (27.4)	502 (26.6)	486 (25.7)
≥25	16470 (37.9)	3425 (40.6)	6909 (40.8)	1160 (33.1)	3269 (34.4)	414 (32.5)	623 (33)	670 (35.4)
Missing	30 (0.1)	12 (0.1)	9 (0.1)	2 (0.1)	3 (0)	1 (0.1)	3 (0.2)	0 (0)
**Smoking**, n (%)								
Non–smoker	8440 (19.4)	8440 (100)	0 (0)	0 (0)	0 (0)	0 (0)	0 (0)	0 (0)
Former smoker	16918 (39)	0 (0)	16918 (100)	0 (0)	0 (0)	0 (0)	0 (0)	0 (0)
1–9 cigarettes/day	3717 (8.6)	0 (0)	0 (0)	3503 (100)	0 (0)	92 (7.2)	78 (4.1)	44 (2.3)
10–20 cigarettes/day	9538 (22)	0 (0)	0 (0)	0 (0)	6693 (70.4)	735 (57.8)	1264 (66.9)	846 (44.8)
21–40 cigarettes/day	4475 (10.3)	0 (0)	0 (0)	0 (0)	2652 (27.9)	420 (33)	524 (27.7)	879 (46.5)
≥40 cigarettes/day	313 (0.7)	0 (0)	0 (0)	0 (0)	167 (1.8)	25 (2)	24 (1.3)	97 (5.1)

**Alcohol drinking frequency**, n (%)								
Non-drinking	14639 (33.7)	3979 (47.1)	6098 (36)	953 (27.2)	2278 (23.9)	366 (28.8)	466 (24.7)	499 (26.4)
1–2 times/week	18878 (43.5)	3188 (37.8)	7193 (42.5)	1759 (50.2)	4578 (48.1)	520 (40.9)	873 (46.2)	767 (40.6)
3–4 times/week	5595 (12.9)	681 (8.1)	1977 (11.7)	446 (12.7)	1615 (17)	205 (16.1)	336 (17.8)	335 (17.7)
≥5 times/week	3889 (9)	498 (5.9)	1460 (8.6)	314 (9)	982 (10.3)	173 (13.6)	204 (10.8)	258 (13.7)
Missing	400 (0.9)	94 (1.1)	190 (1.1)	31 (0.9)	59 (0.6)	8 (0.6)	11 (0.6)	7 (0.4)
**Physical exercise**, n (%)								
0–2 days/week	32986 (76)	6238 (73.9)	12283 (72.6)	2662 (76)	7680 (80.7)	1059 (83.3)	1542 (81.6)	1522 (80.5)
≥3 days/week	9497 (21.9)	2027 (24)	4205 (24.9)	769 (22)	1682 (17.7)	186 (14.6)	314 (16.6)	314 (16.6)
Missing	918 (2.1)	175 (2.1)	430 (2.5)	72 (2.1)	150 (1.6)	27 (2.1)	34 (1.8)	30 (1.6)
**Charlson Comorbidity Index**, n (%)								
0	295 (0.7)	64 (0.8)	111 (0.7)	24 (0.7)	44 (0.5)	18 (1.4)	12 (0.6)	22 (1.2)
1	335 (0.8)	75 (0.9)	130 (0.8)	28 (0.8)	52 (0.5)	17 (1.3)	16 (0.8)	17 (0.9)
2	10301 (23.7)	2043 (24.2)	3989 (23.6)	809 (23.1)	2289 (24.1)	295 (23.2)	410 (21.7)	466 (24.7)
≥3	32470 (74.8)	6258 (74.1)	12688 (75)	2642 (75.4)	7127 (74.9)	942 (74.1)	1452 (76.8)	1361 (72)

[a] number of participants;
[b] five groups of the smoking trajectory model for pre-diagnosis current smokers (n=18043) in the three-measurement subpopulation;
[c] standard deviation.

Supplemental Table S4. Number of cancer cases and death cases

**Cancer types**	**ICD-10**[a]	**Cancer cases**	**Death cases**
Lip, oral cavity, and pharynx	C00-14	2589 (2.3)	263 (1.1)
Esophagus	C15	1304 (1.2)	404 (1.7)
Stomach	C16	31150 (28.2)	1879 (7.9)
Colon and rectum	C18-20	21069 (19.1)	1747 (7.3)
Liver	C22	7068 (6.4)	2930 (12.3)
Gallbladder and biliary tract	C23-24	1547 (1.4)	693 (2.9)
Pancreas	C25	687 (0.6)	614 (2.6)
Larynx	C32	1464 (1.3)	134 (0.6)
Lung	C33-34	6707 (6.1)	3493 (14.6)
Breast	C50	106 (0.1)	9 (0)
Prostate	C61	13138 (11.9)	1137 (4.8)
Testis	C62	94 (0.1)	0 (0)
Kidney	C64	2934 (2.7)	237 (1)
Bladder	C67	4580 (4.1)	447 (1.9)
Brain	C70-72	636 (0.6)	143 (0.6)
Thyroid gland	C73	6721 (6.1)	66 (0.3)
Hodgkin lymphoma	C81	92 (0.1)	9 (0)
Non-Hodgkin lymphoma	C82-86,96	1639 (1.5)	352 (1.5)
Multiple myeloma, malignant plasma cell neoplasms	C90	437 (0.4)	265 (1.1)
Leukemia	C91-95	676 (0.6)	289 (1.2)
Other cancer types	-	5917 (5.4)	761 (3.2)
Other causes of death	-	-	8016 (33.6)
Total	C00-97	110555	23888

[a] International Classification of Diseases, 10th revision.

Supplemental Table S5. All-cause mortality risk according to smoking trajectories for some specific cancers (except gastric, colorectal, liver, and lung cancers)

Smoking trajectories	aHR[a] (95% CI[b])	Overall survival < 5 years	Overall survival ≥ 5 years
**SMOKING-RELATED CANCERS**			
**Lip, oral cavity, and pharyngeal cancer**			
Non-smokers	Reference	Reference	Reference
Former smokers	1.07 (0.90, 1.28)	0.84 (0.56, 1.24)	1.10 (0.90, 1.35)
Light smoking quitters	1.35 (1.07, 1.71)	1.66 (0.97, 2.83)	1.37 (1.04, 1.79)
Heavy smoking quitters	1.32 (1.08, 1.60)	0.86 (0.55, 1.36)	1.40 (1.13, 1.75)
Consistent moderate smokers	1.44 (1.04, 1.99)	1.14 (0.51, 2.52)	1.56 (1.09, 2.23)
Decreasing heavy smokers	1.39 (0.92, 2.09)	1.59 (0.46, 5.42)	1.61 (1.03, 2.51)
**Esophageal cancer**			
Non-smokers	Reference	Reference	Reference
Former smokers	0.88 (0.65, 1.19)	0.62 (0.37, 1.03)	0.98 (0.67, 1.44)
Light smoking quitters	1.23 (0.86, 1.75)	0.98 (0.53, 1.81)	1.38 (0.88, 2.15)
Heavy smoking quitters	1.26 (0.93, 1.71)	0.93 (0.55, 1.55)	1.40 (0.96, 2.06)
Consistent moderate smokers	1.86 (0.97, 3.60)	0.37 (0.12, 1.12)	2.18 (0.94, 5.04)
Decreasing heavy smokers	1.14 (0.52, 2.54)	-	1.76 (0.77, 4.03)
**Pancreatic cancer**			
Non-smokers	Reference	Reference	Reference
Former smokers	0.71 (0.50, 1.01)	0.42 (0.20, 0.88)	0.58 (0.38, 0.88)
Light smoking quitters	0.77 (0.45, 1.33)	0.62 (0.22, 1.71)	0.57 (0.29, 1.12)
Heavy smoking quitters	1.32 (0.91, 1.92)	0.51 (0.23, 1.10)	1.18 (0.74, 1.87)
Consistent moderate smokers	1.34 (0.75, 2.37)	0.23 (0.07, 0.72)	1.23 (0.59, 2.54)
Decreasing heavy smokers	1.44 (0.75, 2.76)	0.88 (0.16, 4.73)	1.39 (0.65, 2.99)
**Laryngeal cancer**			
Non-smokers	Reference	Reference	Reference
Former smokers	1.09 (0.77, 1.54)	0.52 (0.25, 1.06)	1.28 (0.85, 1.91)
Light smoking quitters	1.26 (0.84, 1.90)	0.82 (0.34, 1.99)	1.39 (0.86, 2.23)
Heavy smoking quitters	1.38 (0.98, 1.93)	0.90 (0.46, 1.74)	1.40 (0.94, 2.08)
Consistent moderate smokers	2.42 (1.42, 4.11)	0.70 (0.18, 2.73)	2.78 (1.54, 5.02)
Decreasing heavy smokers	1.57 (0.87, 2.82)	1.03 (0.33, 3.26)	1.30 (0.63, 2.69)
**Kidney cancer**			
Non-smokers	Reference	Reference	Reference
Former smokers	1.00 (0.77, 1.30)	0.79 (0.47, 1.35)	1.02 (0.75, 1.39)
Light smoking quitters	1.64 (1.13, 2.37)	0.51 (0.17, 1.50)	2.15 (1.44, 3.22)
Heavy smoking quitters	1.65 (1.24, 2.20)	1.27 (0.70, 2.28)	1.85 (1.33, 2.59)
Consistent moderate smokers	1.22 (0.72, 2.09)	1.22 (0.41, 3.62)	1.26 (0.68, 2.34)
Decreasing heavy smokers	1.93 (1.06, 3.51)	1.67 (0.46, 6.02)	2.17 (1.09, 4.30)
**Bladder cancer**			
Non-smokers	Reference	Reference	Reference
Former smokers	0.89 (0.74, 1.06)	0.75 (0.53, 1.08)	0.89 (0.73, 1.09)

Light smoking quitters	1.08 (0.85, 1.35)	1.06 (0.68, 1.66)	1.03 (0.79, 1.35)
Heavy smoking quitters	1.20 (0.99, 1.45)	0.99 (0.67, 1.48)	1.27 (1.02, 1.58)
Consistent moderate smokers	1.22 (0.91, 1.62)	1.29 (0.54, 3.10)	1.38 (1.01, 1.88)
Decreasing heavy smokers	1.65 (1.17, 2.32)	3.20 (1.60, 6.39)	1.56 (1.05, 2.33)
**Leukemia**			
Non-smokers	Reference	Reference	Reference
Former smokers	0.69 (0.46, 1.04)	0.49 (0.28, 0.87)	0.97 (0.52, 1.80)
Light smoking quitters	1.63 (0.96, 2.78)	2.72 (1.22, 6.08)	1.96 (0.87, 4.41)
Heavy smoking quitters	0.69 (0.43, 1.10)	0.74 (0.37, 1.50)	1.10 (0.55, 2.18)
Consistent moderate smokers	0.83 (0.25, 2.71)	0.46 (0.05, 3.97)	1.65 (0.36, 7.46)
Decreasing heavy smokers	1.08 (0.31, 3.79)	3.99 (0.55, 29.13)	0.45 (0.05, 4.00)
**CANCERS UNRELATED TO SMOKING**			
**Gallbladder and biliary tract cancer**			
Non-smokers	Reference	Reference	Reference
Former smokers	0.89 (0.72, 1.11)	0.85 (0.60, 1.2)	0.91 (0.69, 1.21)
Light smoking quitters	0.82 (0.58, 1.16)	1.05 (0.63, 1.77)	0.60 (0.36, 0.99)
Heavy smoking quitters	1.15 (0.89, 1.48)	1.22 (0.82, 1.81)	1.06 (0.75, 1.50)
Consistent moderate smokers	1.19 (0.71, 2.02)	1.17 (0.49, 2.79)	1.24 (0.63, 2.41)
Decreasing heavy smokers	0.68 (0.31, 1.46)	1.12 (0.32, 3.86)	0.57 (0.21, 1.60)
**Prostate cancer**			
Non-smokers	Reference	Reference	Reference
Former smokers	1.07 (0.97, 1.18)	0.92 (0.76, 1.11)	1.05 (0.94, 1.18)
Light smoking quitters	1.56 (1.36, 1.80)	1.30 (0.98, 1.72)	1.62 (1.38, 1.91)
Heavy smoking quitters	1.45 (1.28, 1.64)	1.21 (0.95, 1.54)	1.42 (1.23, 1.64)
Consistent moderate smokers	1.98 (1.60, 2.45)	1.64 (1.05, 2.55)	1.96 (1.53, 2.51)
Decreasing heavy smokers	2.06 (1.54, 2.74)	1.79 (1.00, 3.19)	2.09 (1.50, 2.92)
**Brain cancer**			
Non-smokers	Reference	Reference	Reference
Former smokers	0.97 (0.66, 1.42)	0.62 (0.31, 1.25)	1.01 (0.63, 1.65)
Light smoking quitters	0.88 (0.45, 1.70)	0.15 (0.04, 0.63)	0.87 (0.38, 2.01)
Heavy smoking quitters	1.11 (0.74, 1.67)	1.09 (0.51, 2.34)	1.12 (0.67, 1.86)
Consistent moderate smokers	0.89 (0.39, 2.00)	0.49 (0.11, 2.28)	0.96 (0.36, 2.54)
Decreasing heavy smokers	1.38 (0.62, 3.08)	2.81 (0.31, 25.58)	1.74 (0.71, 4.25)
**Thyroid cancer**			
Non-smokers	Reference	Reference	Reference
Former smokers	1.43 (1.01, 2.02)	0.91 (0.43, 1.93)	1.46 (0.99, 2.16)
Light smoking quitters	1.46 (0.87, 2.43)	1.37 (0.46, 4.06)	1.42 (0.79, 2.54)
Heavy smoking quitters	2.23 (1.52, 3.26)	1.75 (0.77, 4.01)	2.23 (1.45, 3.45)
Consistent moderate smokers	1.65 (0.83, 3.29)	1.44 (0.38, 5.53)	1.56 (0.69, 3.53)
Decreasing heavy smokers	1.96 (0.95, 4.01)	4.45 (1.16, 17.09)	1.62 (0.68, 3.86)
**Non-Hodgkin lymphoma**			
Non-smokers	Reference	Reference	Reference

Former smokers	0.80 (0.59, 1.08)	0.51 (0.29, 0.9)	0.91 (0.63, 1.30)
Light smoking quitters	0.70 (0.42, 1.15)	1.34 (0.57, 3.19)	0.60 (0.31, 1.14)
Heavy smoking quitters	1.08 (0.78, 1.51)	0.56 (0.30, 1.06)	1.13 (0.75, 1.69)
Consistent moderate smokers	0.70 (0.30, 1.61)	0.39 (0.08, 1.87)	0.68 (0.24, 1.92)
Decreasing heavy smokers	0.70 (0.28, 1.76)	-	1.12 (0.44, 2.86)
**Multiple myeloma and malignant plasma cell neoplasms**			
Non-smokers	Reference	Reference	Reference
Former smokers	1.06 (0.74, 1.51)	0.64 (0.37, 1.11)	1.23 (0.77, 1.98)
Light smoking quitters	1.99 (1.18, 3.36)	1.46 (0.67, 3.18)	2.00 (0.92, 4.35)
Heavy smoking quitters	1.07 (0.70, 1.65)	0.73 (0.36, 1.45)	1.21 (0.68, 2.13)
Consistent moderate smokers	1.18 (0.45, 3.08)	3.98 (1.03, 15.39)	0.55 (0.12, 2.48)
Decreasing heavy smokers	3.67 (1.06, 12.75)	1.34 (0.38, 4.76)	1.15 (0.10, 12.82)

[a] adjusted hazard ratio, adjusted for age at cancer diagnosis, income, body mass index, alcohol drinking, physical activity, and Charlson Comorbidity Index;
[b] confidence interval.

Supplemental Table S6. Mortality risk according to smoking trajectories for pooled cancer groups and liver and lung cancers in the three-measurement subpopulation

Smoking trajectories	aHR[a] (95% CI[b])		
	Overall	Subgroup: Overall survival <5 years	Subgroup: Overall survival ≥5 years
**SMOKING-RELATED CANCERS**			
**All-cause mortality**			
Non-smokers	Reference	Reference	Reference
Former smokers	1.12 (1.04, 1.22)	1.00 (0.81, 1.23)	1.16 (1.06, 1.27)
Light smoking quitters	1.42 (1.27, 1.59)	0.93 (0.69, 1.25)	1.51 (1.34, 1.70)
Heavy smoking quitters	1.53 (1.40, 1.68)	1.12 (0.90, 1.41)	1.58 (1.43, 1.75)
Late heavy-smoking quitters	1.57 (1.34, 1.84)	1.17 (0.70, 1.94)	1.73 (1.47, 2.05)
Heavy smoking relapse	1.67 (1.46, 1.91)	1.05 (0.73, 1.53)	1.79 (1.55, 2.07)
Consistent heavy smokers	1.57 (1.36, 1.82)	0.81 (0.55, 1.22)	1.67 (1.43, 1.96)
**Cancer mortality**			
Non-smokers	Reference	Reference	Reference
Former smokers	1.12 (1.01, 1.25)	0.92 (0.73, 1.17)	1.19 (1.05, 1.34)
Light smoking quitters	1.52 (1.32, 1.75)	0.93 (0.67, 1.30)	1.65 (1.42, 1.93)
Heavy smoking quitters	1.58 (1.41, 1.77)	1.13 (0.87, 1.45)	1.65 (1.45, 1.88)
Late heavy-smoking quitters	1.63 (1.34, 1.99)	1.01 (0.56, 1.84)	1.89 (1.53, 2.33)
Heavy smoking relapse	1.68 (1.42, 2.00)	0.81 (0.52, 1.28)	1.93 (1.60, 2.32)
Consistent heavy smokers	1.70 (1.42, 2.03)	0.74 (0.47, 1.17)	1.90 (1.56, 2.30)
**CANCERS UNRELATED TO SMOKING**			
**All-cause mortality**			
Non-smokers	Reference	Reference	Reference
Former smokers	1.10 (0.97, 1.24)	0.79 (0.54, 1.14)	1.10 (0.97, 1.26)
Light smoking quitters	1.28 (1.06, 1.55)	1.29 (0.76, 2.18)	1.25 (1.01, 1.54)
Heavy smoking quitters	1.35 (1.15, 1.60)	0.87 (0.54, 1.40)	1.36 (1.14, 1.63)
Late heavy-smoking quitters	1.64 (1.27, 2.12)	1.05 (0.52, 2.12)	1.59 (1.21, 2.10)
Heavy smoking relapse	1.62 (1.24, 2.11)	0.62 (0.29, 1.31)	1.62 (1.21, 2.15)
Consistent heavy smokers	1.78 (1.37, 2.31)	1.11 (0.51, 2.41)	1.82 (1.38, 2.41)
**Cancer mortality**			
Non-smokers	Reference	Reference	Reference
Former smokers	1.20 (1.02, 1.42)	0.63 (0.40, 0.97)	1.27 (1.06, 1.52)
Light smoking quitters	1.52 (1.19, 1.93)	1.47 (0.82, 2.61)	1.46 (1.11, 1.91)
Heavy smoking quitters	1.48 (1.20, 1.82)	0.71 (0.40, 1.25)	1.56 (1.24, 1.96)
Late heavy-smoking quitters	1.66 (1.20, 2.31)	1.22 (0.57, 2.59)	1.56 (1.08, 2.26)
Heavy smoking relapse	1.50 (1.06, 2.13)	0.46 (0.18, 1.18)	1.55 (1.06, 2.27)
Consistent heavy smokers	1.89 (1.38, 2.59)	1.01 (0.41, 2.48)	1.99 (1.42, 2.80)
**LIVER CANCER**			
**All-cause mortality**			
Non-smokers	Reference	Reference	Reference

Former smokers	1.04 (0.85, 1.27)	1.02 (0.65, 1.60)	1.07 (0.85, 1.34)
Light smoking quitters	1.14 (0.87, 1.50)	1.29 (0.72, 2.32)	1.10 (0.80, 1.51)
Heavy smoking quitters	1.11 (0.89, 1.39)	1.35 (0.80, 2.26)	1.05 (0.81, 1.37)
Late heavy-smoking quitters	1.09 (0.74, 1.62)	1.42 (0.55, 3.65)	1.09 (0.70, 1.69)
Heavy smoking relapse	1.27 (0.91, 1.77)	2.50 (1.00, 6.24)	1.42 (0.99, 2.04)
Consistent heavy smokers	1.23 (0.87, 1.75)	0.95 (0.36, 2.53)	1.34 (0.92, 1.98)
**Cancer mortality**			
Non-smokers	Reference	Reference	Reference
Former smokers	1.07 (0.86, 1.33)	1.06 (0.66, 1.69)	1.11 (0.86, 1.42)
Light smoking quitters	1.14 (0.85, 1.53)	1.26 (0.68, 2.32)	1.11 (0.79, 1.57)
Heavy smoking quitters	1.09 (0.86, 1.39)	1.36 (0.79, 2.32)	1.03 (0.78, 1.37)
Late heavy-smoking quitters	1.04 (0.68, 1.61)	0.98 (0.32, 2.99)	1.13 (0.70, 1.82)
Heavy smoking relapse	1.22 (0.85, 1.75)	2.31 (0.85, 6.23)	1.41 (0.95, 2.10)
Consistent heavy smokers	1.21 (0.83, 1.76)	0.81 (0.28, 2.33)	1.37 (0.90, 2.07)
**Cancer-specific mortality**			
Non-smokers	Reference	Reference	Reference
Former smokers	1.08 (0.85, 1.36)	1.07 (0.66, 1.73)	1.12 (0.85, 1.47)
Light smoking quitters	1.11 (0.80, 1.53)	1.25 (0.67, 2.35)	1.06 (0.72, 1.56)
Heavy smoking quitters	1.07 (0.82, 1.39)	1.24 (0.71, 2.17)	1.02 (0.75, 1.39)
Late heavy-smoking quitters	0.90 (0.54, 1.49)	0.76 (0.21, 2.66)	1.00 (0.57, 1.76)
Heavy smoking relapse	1.22 (0.83, 1.81)	2.44 (0.90, 6.64)	1.43 (0.93, 2.20)
Consistent heavy smokers	0.97 (0.62, 1.51)	0.88 (0.30, 2.53)	1.05 (0.63, 1.73)
**LUNG CANCER**			
**All-cause mortality**			
Non-smokers	Reference	Reference	Reference
Former smokers	0.89 (0.69, 1.17)	1.58 (0.87, 2.87)	1.04 (0.77, 1.42)
Light smoking quitters	1.14 (0.81, 1.60)	0.48 (0.19, 1.21)	1.38 (0.94, 2.02)
Heavy smoking quitters	1.08 (0.83, 1.40)	1.34 (0.70, 2.57)	1.34 (0.99, 1.82)
Late heavy-smoking quitters	1.46 (0.89, 2.38)	3.20 (0.47, 21.9)	1.87 (1.10, 3.18)
Heavy smoking relapse	1.51 (0.96, 2.36)	1.30 (0.55, 3.04)	1.34 (0.77, 2.35)
Consistent heavy smokers	0.90 (0.52, 1.56)	0.74 (0.16, 3.42)	1.12 (0.62, 2.03)
**Cancer mortality**			
Non-smokers	Reference	Reference	Reference
Former smokers	0.77 (0.58, 1.03)	1.38 (0.73, 2.61)	0.89 (0.63, 1.26)
Light smoking quitters	0.98 (0.67, 1.44)	0.50 (0.19, 1.32)	1.17 (0.75, 1.81)
Heavy smoking quitters	0.95 (0.71, 1.26)	1.25 (0.62, 2.49)	1.17 (0.84, 1.64)
Late heavy-smoking quitters	1.45 (0.86, 2.47)	6.03 (0.99, 36.89)	1.84 (1.03, 3.28)
Heavy smoking relapse	1.32 (0.80, 2.19)	1.06 (0.41, 2.76)	1.26 (0.68, 2.35)
Consistent heavy smokers	0.83 (0.45, 1.51)	0.43 (0.05, 3.46)	1.09 (0.57, 2.08)
**Cancer-specific mortality**			
Non-smokers	Reference	Reference	Reference
Former smokers	0.89 (0.63, 1.27)	1.85 (0.93, 3.70)	1.03 (0.67, 1.58)
Light smoking quitters	1.20 (0.77, 1.88)	0.58 (0.20, 1.65)	1.49 (0.88, 2.53)

Heavy smoking quitters	1.17 (0.83, 1.64)	1.50 (0.71, 3.16)	1.52 (1.00, 2.30)
Late heavy-smoking quitters	1.40 (0.72, 2.73)	6.25 (0.70, 55.45)	1.93 (0.93, 4.01)
Heavy smoking relapse	1.75 (1.00, 3.08)	1.64 (0.60, 4.48)	1.62 (0.79, 3.36)
Consistent heavy smokers	0.58 (0.25, 1.38)	-	0.89 (0.36, 2.16)

[a] adjusted hazard ratio, adjusted for age at cancer diagnosis, income, body mass index, alcohol drinking, physical activity, and Charlson Comorbidity Index;
[b] confidence interval.

Supplemental Table S7. Mortality risk according to the smoking status for pooled cancers, pooled cancer groups, and four leading cancers (gastric, colorectal, liver, and lung cancers)

Smoking status	aHR[a] (95% CI[b])		
	Overall	Subgroup: Overall survival <5 years	Subgroup: Overall survival ≥5 years
**ALL CANCERS**			
**All-cause mortality**			
Non-smokers	Reference	Reference	Reference
Former smokers	1.04 (1.00, 1.08)	0.78 (0.73, 0.83)	1.08 (1.04, 1.13)
Light smoking quitters	1.35 (1.31, 1.41)	1.09 (1.02, 1.16)	1.39 (1.32, 1.45)
Heavy smoking quitters	1.44 (1.38, 1.51)	0.98 (0.90, 1.07)	1.59 (1.51, 1.67)
**Cancer mortality**			
Non-smokers	Reference	Reference	Reference
Former smokers	1.04 (1.00, 1.09)	0.77 (0.72, 0.83)	1.10 (1.04, 1.17)
Light smoking quitters	1.41 (1.35, 1.48)	1.11 (1.03, 1.19)	1.47 (1.38, 1.56)
Heavy smoking quitters	1.40 (1.32, 1.48)	0.90 (0.82, 1.00)	1.62 (1.52, 1.74)
**SMOKING-RELATED CANCERS**			
**All-cause mortality**			
Non-smokers	Reference	Reference	Reference
Former smokers	1.03 (0.99, 1.07)	0.76 (0.70, 0.82)	1.08 (1.03, 1.14)
Light smoking quitters	1.33 (1.27, 1.39)	1.02 (0.94, 1.10)	1.37 (1.30, 1.45)
Heavy smoking quitters	1.40 (1.33, 1.48)	0.91 (0.83, 1.01)	1.57 (1.48, 1.67)
**Cancer mortality**			
Non-smokers	Reference	Reference	Reference
Former smokers	1.02 (0.97, 1.08)	0.74 (0.68, 0.81)	1.10 (1.03, 1.18)
Light smoking quitters	1.36 (1.29, 1.44)	1.01 (0.93, 1.10)	1.45 (1.36, 1.55)
Heavy smoking quitters	1.36 (1.27, 1.45)	0.82 (0.73, 0.91)	1.64 (1.51, 1.77)
**CANCERS UNRELATED TO SMOKING**			
**All-cause mortality**			
Non-smokers	Reference	Reference	Reference
Former smokers	1.05 (0.98, 1.12)	0.81 (0.72, 0.92)	1.07 (0.99, 1.16)
Light smoking quitters	1.37 (1.26, 1.48)	1.16 (1.01, 1.34)	1.39 (1.27, 1.53)
Heavy smoking quitters	1.53 (1.40, 1.68)	1.13 (0.95, 1.34)	1.62 (1.46, 1.81)
**Cancer mortality**			
Non-smokers	Reference	Reference	Reference
Former smokers	1.07 (0.98, 1.16)	0.82 (0.71, 0.94)	1.10 (0.99, 1.22)
Light smoking quitters	1.48 (1.35, 1.63)	1.28 (1.09, 1.50)	1.50 (1.33, 1.69)
Heavy smoking quitters	1.47 (1.31, 1.65)	1.12 (0.92, 1.37)	1.56 (1.36, 1.79)
**GASTRIC CANCER**			
**All-cause mortality**			
Non-smokers	Reference	Reference	Reference
Former smokers	1.03 (0.95, 1.11)	0.76 (0.66, 0.89)	1.08 (0.99, 1.18)

Light smoking quitters	1.38 (1.27, 1.49)	1.04 (0.9, 1.21)	1.42 (1.29, 1.56)
Heavy smoking quitters	1.64 (1.50, 1.79)	1.00 (0.84, 1.2)	1.79 (1.61, 1.99)
**Cancer mortality**			
Non-smokers	Reference	Reference	Reference
Former smokers	1.06 (0.95, 1.18)	0.76 (0.64, 0.91)	1.16 (1.01, 1.34)
Light smoking quitters	1.51 (1.35, 1.69)	1.05 (0.87, 1.25)	1.65 (1.44, 1.90)
Heavy smoking quitters	1.71 (1.51, 1.94)	0.85 (0.68, 1.07)	2.14 (1.83, 2.50)
**Cancer-specific mortality**			
Non-smokers	Reference	Reference	Reference
Former smokers	0.91 (0.78, 1.06)	0.73 (0.59, 0.90)	0.97 (0.78, 1.21)
Light smoking quitters	1.17 (1.00, 1.37)	0.94 (0.76, 1.16)	1.19 (0.95, 1.49)
Heavy smoking quitters	1.08 (0.89, 1.30)	0.73 (0.56, 0.96)	1.24 (0.95, 1.63)
**COLORECTAL CANCER**			
**All-cause mortality**			
Non-smokers	Reference	Reference	Reference
Former smokers	1.07 (0.98, 1.17)	0.73 (0.62, 0.86)	1.12 (1.01, 1.24)
Light smoking quitters	1.29 (1.17, 1.43)	0.93 (0.78, 1.11)	1.35 (1.19, 1.52)
Heavy smoking quitters	1.38 (1.23, 1.55)	0.80 (0.64, 0.99)	1.59 (1.38, 1.82)
**Cancer mortality**			
Non-smokers	Reference	Reference	Reference
Former smokers	1.10 (0.98, 1.24)	0.69 (0.57, 0.82)	1.23 (1.06, 1.43)
Light smoking quitters	1.37 (1.21, 1.55)	0.88 (0.73, 1.08)	1.54 (1.31, 1.81)
Heavy smoking quitters	1.34 (1.15, 1.55)	0.66 (0.50, 0.85)	1.74 (1.45, 2.09)
**Cancer-specific mortality**			
Non-smokers	Reference	Reference	Reference
Former smokers	0.99 (0.86, 1.15)	0.64 (0.52, 0.78)	1.15 (0.93, 1.43)
Light smoking quitters	1.17 (1.00, 1.38)	0.80 (0.64, 0.99)	1.35 (1.07, 1.70)
Heavy smoking quitters	0.77 (0.62, 0.95)	0.49 (0.36, 0.67)	0.94 (0.70, 1.28)
**LIVER CANCER**			
**All-cause mortality**			
Non-smokers	Reference	Reference	Reference
Former smokers	1.06 (0.96, 1.18)	0.78 (0.66, 0.92)	1.15 (1.01, 1.32)
Light smoking quitters	1.13 (1.01, 1.26)	0.94 (0.79, 1.12)	1.12 (0.97, 1.29)
Heavy smoking quitters	1.23 (1.08, 1.39)	0.99 (0.80, 1.22)	1.34 (1.14, 1.57)
**Cancer mortality**			
Non-smokers	Reference	Reference	Reference
Former smokers	1.04 (0.93, 1.17)	0.80 (0.67, 0.95)	1.11 (0.96, 1.29)
Light smoking quitters	1.09 (0.97, 1.22)	0.93 (0.77, 1.11)	1.07 (0.92, 1.25)
Heavy smoking quitters	1.17 (1.02, 1.34)	0.98 (0.79, 1.22)	1.27 (1.06, 1.51)
**Cancer-specific mortality**			
Non-smokers	Reference	Reference	Reference
Former smokers	1.04 (0.92, 1.17)	0.79 (0.66, 0.95)	1.11 (0.95, 1.31)

Light smoking quitters	1.05 (0.92, 1.19)	0.91 (0.75, 1.10)	1.01 (0.85, 1.19)
Heavy smoking quitters	1.09 (0.94, 1.27)	0.96 (0.77, 1.21)	1.14 (0.94, 1.39)
**LUNG CANCER**			
**All-cause mortality**			
Non-smokers	Reference	Reference	Reference
Former smokers	1.01 (0.89, 1.15)	0.89 (0.73, 1.09)	1.09 (0.92, 1.29)
Light smoking quitters	1.14 (1.01, 1.30)	1.08 (0.89, 1.30)	1.24 (1.05, 1.46)
Heavy smoking quitters	1.28 (1.09, 1.51)	1.08 (0.82, 1.43)	1.53 (1.24, 1.88)
**Cancer mortality**			
Non-smokers	Reference	Reference	Reference
Former smokers	0.97 (0.84, 1.12)	0.85 (0.69, 1.05)	1.07 (0.87, 1.30)
Light smoking quitters	1.08 (0.94, 1.23)	1.04 (0.86, 1.28)	1.15 (0.95, 1.39)
Heavy smoking quitters	1.14 (0.95, 1.38)	0.98 (0.73, 1.31)	1.40 (1.1, 1.79)
**Cancer-specific mortality**			
Non-smokers	Reference	Reference	Reference
Former smokers	1.07 (0.91, 1.26)	0.87 (0.70, 1.08)	1.33 (1.04, 1.71)
Light smoking quitters	1.23 (1.05, 1.44)	1.07 (0.86, 1.32)	1.54 (1.21, 1.96)
Heavy smoking quitters	1.11 (0.89, 1.38)	0.99 (0.72, 1.35)	1.46 (1.07, 2.00)

[a] adjusted hazard ratio, adjusted for age at cancer diagnosis, income, body mass index, alcohol drinking, physical activity, and Charlson Comorbidity Index;
[b] confidence interval.

Supplemental Table S8. Mortality risk according to the pre-diagnosis smoking levels for pooled cancers, pooled cancer groups, and four leading cancers (gastric, colorectal, liver, and lung cancers)

Pre-diagnosis smoking levels	aHR[a] (95% CI[b])		
	Overall	Subgroup: Overall survival <5 years	Subgroup: Overall survival ≥5 years
**ALL CANCERS**			
**All-cause mortality**			
Non–smoker	Reference	Reference	Reference
Former smoker	1.04 (1.00, 1.08)	0.78 (0.73, 0.83)	1.08 (1.04, 1.13)
1–9 cigarettes/day	1.34 (1.28, 1.40)	1.11 (1.02, 1.21)	1.38 (1.30, 1.46)
10–20 cigarettes/day	1.39 (1.33, 1.44)	0.98 (0.91, 1.05)	1.45 (1.39, 1.53)
21–40 cigarettes/day	1.43 (1.36, 1.51)	1.21 (1.11, 1.33)	1.54 (1.45, 1.64)
≥40 cigarettes/day	1.35 (1.15, 1.58)	1.53 (1.13, 2.05)	1.47 (1.21, 1.77)
**Cancer mortality**			
Non–smoker	Reference	Reference	Reference
Former smoker	1.04 (1.00, 1.09)	0.77 (0.72, 0.83)	1.10 (1.04, 1.17)
1–9 cigarettes/day	1.37 (1.29, 1.46)	1.09 (0.99, 1.20)	1.45 (1.35, 1.57)
10–20 cigarettes/day	1.41 (1.34, 1.48)	0.97 (0.90, 1.05)	1.51 (1.42, 1.61)
21–40 cigarettes/day	1.44 (1.35, 1.53)	1.19 (1.07, 1.32)	1.61 (1.49, 1.74)
≥40 cigarettes/day	1.34 (1.11, 1.62)	1.49 (1.08, 2.07)	1.50 (1.19, 1.89)
**SMOKING-RELATED CANCERS**			
**All-cause mortality**			
Non–smoker	Reference	Reference	Reference
Former smoker	1.03 (0.99, 1.07)	0.76 (0.70, 0.82)	1.08 (1.03, 1.14)
1–9 cigarettes/day	1.31 (1.24, 1.39)	1.03 (0.94, 1.14)	1.37 (1.28, 1.46)
10–20 cigarettes/day	1.35 (1.29, 1.42)	0.91 (0.84, 0.99)	1.44 (1.36, 1.52)
21–40 cigarettes/day	1.40 (1.32, 1.49)	1.14 (1.03, 1.27)	1.52 (1.42, 1.63)
≥40 cigarettes/day	1.31 (1.10, 1.57)	1.58 (1.14, 2.18)	1.45 (1.18, 1.80)
**Cancer mortality**			
Non–smoker	Reference	Reference	Reference
Former smoker	1.02 (0.97, 1.08)	0.74 (0.68, 0.81)	1.10 (1.03, 1.18)
1–9 cigarettes/day	1.33 (1.24, 1.42)	0.98 (0.88, 1.10)	1.44 (1.32, 1.57)
10–20 cigarettes/day	1.36 (1.29, 1.44)	0.88 (0.81, 0.97)	1.51 (1.41, 1.62)
21–40 cigarettes/day	1.40 (1.30, 1.50)	1.10 (0.98, 1.24)	1.59 (1.46, 1.74)
≥40 cigarettes/day	1.29 (1.05, 1.59)	1.50 (1.05, 2.15)	1.50 (1.16, 1.93)
**CANCERS UNRELATED TO SMOKING**			
**All-cause mortality**			
Non–smoker	Reference	Reference	Reference
Former smoker	1.05 (0.98, 1.12)	0.81 (0.72, 0.92)	1.07 (0.99, 1.16)
1–9 cigarettes/day	1.37 (1.24, 1.51)	1.24 (1.04, 1.48)	1.41 (1.26, 1.59)
10–20 cigarettes/day	1.44 (1.33, 1.57)	1.08 (0.93, 1.26)	1.47 (1.33, 1.62)
21–40 cigarettes/day	1.46 (1.30, 1.64)	1.22 (0.98, 1.53)	1.58 (1.38, 1.81)

≥40 cigarettes/day	1.37 (0.95, 2.00)	1.17 (0.55, 2.47)	1.45 (0.94, 2.24)
**Cancer mortality**			
Non–smoker	Reference	Reference	Reference
Former smoker	1.07 (0.98, 1.16)	0.82 (0.71, 0.94)	1.10 (0.99, 1.22)
1–9 cigarettes/day	1.46 (1.29, 1.64)	1.37 (1.12, 1.67)	1.48 (1.28, 1.72)
10–20 cigarettes/day	1.49 (1.34, 1.65)	1.16 (0.98, 1.37)	1.49 (1.31, 1.69)
21–40 cigarettes/day	1.49 (1.30, 1.71)	1.21 (0.94, 1.57)	1.67 (1.41, 1.97)
≥40 cigarettes/day	1.41 (0.90, 2.20)	1.25 (0.55, 2.82)	1.46 (0.86, 2.49)
**GASTRIC CANCER**			
**All-cause mortality**			
Non–smoker	Reference	Reference	Reference
Former smoker	1.03 (0.95, 1.11)	0.76 (0.66, 0.89)	1.08 (0.99, 1.19)
1–9 cigarettes/day	1.37 (1.25, 1.52)	1.06 (0.88, 1.28)	1.41 (1.25, 1.58)
10–20 cigarettes/day	1.48 (1.37, 1.61)	0.97 (0.83, 1.14)	1.56 (1.42, 1.72)
21–40 cigarettes/day	1.53 (1.38, 1.71)	1.14 (0.92, 1.41)	1.66 (1.47, 1.89)
≥40 cigarettes/day	1.35 (0.96, 1.89)	1.63 (0.77, 3.47)	1.57 (1.08, 2.30)
**Cancer mortality**			
Non–smoker	Reference	Reference	Reference
Former smoker	1.06 (0.95, 1.18)	0.76 (0.64, 0.92)	1.16 (1.01, 1.34)
1–9 cigarettes/day	1.47 (1.28, 1.69)	0.99 (0.78, 1.24)	1.65 (1.39, 1.96)
10–20 cigarettes/day	1.60 (1.43, 1.80)	0.92 (0.76, 1.12)	1.85 (1.60, 2.14)
21–40 cigarettes/day	1.64 (1.42, 1.89)	1.15 (0.90, 1.48)	1.91 (1.60, 2.29)
≥40 cigarettes/day	1.49 (0.97, 2.30)	2.14 (1.00, 4.58)	1.77 (1.05, 2.98)
**Cancer-specific mortality**			
Non–smoker	Reference	Reference	Reference
Former smoker	0.91 (0.78, 1.06)	0.73 (0.59, 0.90)	0.97 (0.78, 1.21)
1–9 cigarettes/day	1.19 (0.98, 1.45)	0.83 (0.63, 1.11)	1.39 (1.06, 1.84)
10–20 cigarettes/day	1.17 (0.99, 1.38)	0.87 (0.69, 1.08)	1.17 (0.92, 1.49)
21–40 cigarettes/day	1.00 (0.80, 1.25)	0.91 (0.67, 1.23)	1.03 (0.75, 1.42)
≥40 cigarettes/day	1.28 (0.71, 2.28)	2.28 (1.00, 5.19)	1.42 (0.62, 3.23)
**COLORECTAL CANCER**			
**All-cause mortality**			
Non–smoker	Reference	Reference	Reference
Former smoker	1.07 (0.98, 1.17)	0.73 (0.62, 0.86)	1.12 (1.01, 1.25)
1–9 cigarettes/day	1.28 (1.13, 1.44)	1.01 (0.81, 1.26)	1.32 (1.13, 1.54)
10–20 cigarettes/day	1.28 (1.15, 1.42)	0.76 (0.63, 0.92)	1.41 (1.24, 1.60)
21–40 cigarettes/day	1.46 (1.27, 1.68)	1.01 (0.78, 1.30)	1.61 (1.36, 1.90)
≥40 cigarettes/day	2.08 (1.42, 3.06)	2.06 (1.06, 4.02)	2.36 (1.47, 3.78)
**Cancer mortality**			
Non–smoker	Reference	Reference	Reference
Former smoker	1.10 (0.99, 1.24)	0.69 (0.57, 0.82)	1.24 (1.06, 1.43)
1–9 cigarettes/day	1.28 (1.09, 1.50)	0.91 (0.71, 1.18)	1.41 (1.15, 1.74)

10–20 cigarettes/day	1.33 (1.16, 1.52)	0.69 (0.55, 0.86)	1.63 (1.37, 1.93)
21–40 cigarettes/day	1.51 (1.27, 1.79)	0.94 (0.71, 1.25)	1.82 (1.47, 2.26)
≥40 cigarettes/day	1.98 (1.24, 3.18)	2.17 (1.07, 4.40)	2.18 (1.16, 4.11)
**Cancer-specific mortality**			
Non–smoker	Reference	Reference	Reference
Former smoker	0.99 (0.86, 1.15)	0.64 (0.52, 0.78)	1.15 (0.93, 1.43)
1–9 cigarettes/day	1.18 (0.97, 1.45)	0.91 (0.69, 1.20)	1.29 (0.95, 1.74)
10–20 cigarettes/day	0.93 (0.78, 1.12)	0.55 (0.43, 0.71)	1.15 (0.89, 1.48)
21–40 cigarettes/day	1.05 (0.84, 1.32)	0.79 (0.57, 1.09)	1.20 (0.87, 1.67)
≥40 cigarettes/day	1.55 (0.85, 2.83)	1.56 (0.64, 3.81)	1.98 (0.87, 4.50)
**LIVER CANCER**			
**All-cause mortality**			
Non–smoker	Reference	Reference	Reference
Former smoker	1.06 (0.96, 1.18)	0.78 (0.66, 0.92)	1.15 (1.01, 1.32)
1–9 cigarettes/day	1.16 (1.01, 1.33)	0.93 (0.75, 1.16)	1.17 (0.98, 1.39)
10–20 cigarettes/day	1.16 (1.03, 1.30)	0.93 (0.78, 1.11)	1.19 (1.03, 1.38)
21–40 cigarettes/day	1.19 (1.03, 1.39)	1.09 (0.85, 1.39)	1.24 (1.02, 1.51)
≥40 cigarettes/day	0.87 (0.49, 1.54)	5.66 (1.39, 23.1)	1.10 (0.58, 2.06)
**Cancer mortality**			
Non–smoker	Reference	Reference	Reference
Former smoker	1.04 (0.93, 1.17)	0.80 (0.67, 0.95)	1.11 (0.96, 1.29)
1–9 cigarettes/day	1.12 (0.97, 1.30)	0.91 (0.72, 1.14)	1.14 (0.94, 1.38)
10–20 cigarettes/day	1.11 (0.98, 1.26)	0.93 (0.77, 1.12)	1.12 (0.95, 1.31)
21–40 cigarettes/day	1.14 (0.97, 1.34)	1.03 (0.80, 1.34)	1.20 (0.97, 1.48)
≥40 cigarettes/day	0.87 (0.48, 1.59)	5.68 (1.39, 23.22)	1.11 (0.57, 2.15)
**Cancer-specific mortality**			
Non–smoker	Reference	Reference	Reference
Former smoker	1.04 (0.92, 1.17)	0.79 (0.66, 0.95)	1.11 (0.95, 1.30)
1–9 cigarettes/day	1.07 (0.91, 1.26)	0.88 (0.69, 1.13)	1.06 (0.86, 1.32)
10–20 cigarettes/day	1.08 (0.94, 1.23)	0.92 (0.75, 1.12)	1.07 (0.90, 1.27)
21–40 cigarettes/day	1.04 (0.87, 1.25)	1.04 (0.79, 1.36)	1.02 (0.81, 1.30)
≥40 cigarettes/day	0.46 (0.19, 1.11)	3.07 (0.43, 22.04)	0.57 (0.21, 1.55)
**LUNG CANCER**			
**All-cause mortality**			
Non–smoker	Reference	Reference	Reference
Former smoker	1.01 (0.89, 1.15)	0.89 (0.73, 1.09)	1.09 (0.92, 1.29)
1–9 cigarettes/day	1.18 (1.01, 1.39)	1.16 (0.90, 1.50)	1.29 (1.04, 1.59)
10–20 cigarettes/day	1.20 (1.05, 1.37)	1.03 (0.84, 1.26)	1.29 (1.08, 1.53)
21–40 cigarettes/day	1.11 (0.95, 1.29)	1.11 (0.86, 1.42)	1.32 (1.09, 1.60)
≥40 cigarettes/day	0.97 (0.66, 1.42)	1.33 (0.73, 2.41)	1.08 (0.66, 1.78)
**Cancer mortality**			
Non–smoker	Reference	Reference	Reference

Former smoker	0.97 (0.84, 1.12)	0.85 (0.69, 1.05)	1.07 (0.87, 1.30)
1–9 cigarettes/day	1.10 (0.91, 1.32)	1.12 (0.86, 1.46)	1.17 (0.91, 1.50)
10–20 cigarettes/day	1.15 (0.99, 1.33)	1.00 (0.81, 1.24)	1.23 (1.01, 1.51)
21–40 cigarettes/day	0.99 (0.84, 1.18)	1.04 (0.80, 1.35)	1.18 (0.94, 1.48)
≥40 cigarettes/day	0.80 (0.51, 1.25)	1.05 (0.53, 2.08)	0.92 (0.51, 1.66)
**Cancer-specific mortality**			
Non–smoker	Reference	Reference	Reference
Former smoker	1.07 (0.91, 1.26)	0.87 (0.70, 1.08)	1.33 (1.04, 1.71)
1–9 cigarettes/day	1.26 (1.03, 1.55)	1.17 (0.89, 1.55)	1.55 (1.14, 2.10)
10–20 cigarettes/day	1.26 (1.07, 1.49)	0.99 (0.79, 1.24)	1.59 (1.23, 2.04)
21–40 cigarettes/day	1.10 (0.91, 1.34)	1.12 (0.86, 1.47)	1.45 (1.10, 1.93)
≥40 cigarettes/day	0.82 (0.49, 1.36)	1.05 (0.51, 2.17)	1.01 (0.49, 2.09)

[a] adjusted hazard ratio, adjusted for age at cancer diagnosis, income, body mass index, alcohol drinking, physical activity, and Charlson Comorbidity Index;
[b] confidence interval.

Supplemental Table S9. Competing risk analysis: Mortality risk according to smoking trajectories for pooled cancers, pooled cancer groups, and four leading cancers (gastric, colorectal, liver, and lung cancers)

Smoking trajectories	aHR[a] (95% CI[b])		
	Overall	Subgroup: Overall survival <5 years	Subgroup: Overall survival ≥5 years
**ALL CANCERS**			
**Cancer mortality**			
Non-smokers	Reference	Reference	Reference
Former smokers	1.03 (0.98, 1.07)	0.78 (0.73, 0.84)	1.08 (1.02, 1.15)
Light smoking quitters	1.32 (1.25, 1.41)	1.06 (0.96, 1.17)	1.39 (1.29, 1.50)
Heavy smoking quitters	1.38 (1.31, 1.45)	1.06 (0.98, 1.14)	1.45 (1.37, 1.54)
Consistent moderate smokers	1.39 (1.28, 1.51)	0.81 (0.69, 0.94)	1.62 (1.46, 1.79)
Decreasing heavy smokers	1.32 (1.19, 1.45)	1.03 (0.85, 1.25)	1.58 (1.41, 1.78)
**SMOKING-RELATED CANCERS**			
**Cancer mortality**			
Non-smokers	Reference	Reference	Reference
Former smokers	1.01 (0.96, 1.06)	0.75 (0.69, 0.82)	1.08 (1.01, 1.16)
Light smoking quitters	1.28 (1.20, 1.38)	0.96 (0.86, 1.07)	1.38 (1.27, 1.51)
Heavy smoking quitters	1.33 (1.26, 1.41)	0.97 (0.89, 1.05)	1.44 (1.34, 1.55)
Consistent moderate smokers	1.35 (1.23, 1.48)	0.71 (0.59, 0.84)	1.64 (1.47, 1.84)
Decreasing heavy smokers	1.27 (1.13, 1.42)	0.97 (0.78, 1.19)	1.57 (1.38, 1.79)
**CANCERS UNRELATED TO SMOKING**			
**Cancer mortality**			
Non-smokers	Reference	Reference	Reference
Former smokers	1.05 (0.96, 1.14)	0.82 (0.71, 0.95)	1.08 (0.97, 1.20)
Light smoking quitters	1.40 (1.24, 1.59)	1.33 (1.09, 1.63)	1.41 (1.20, 1.64)
Heavy smoking quitters	1.44 (1.31, 1.59)	1.20 (1.02, 1.42)	1.47 (1.30, 1.66)
Consistent moderate smokers	1.48 (1.23, 1.77)	1.13 (0.82, 1.56)	1.51 (1.21, 1.87)
Decreasing heavy smokers	1.43 (1.16, 1.77)	1.18 (0.77, 1.82)	1.61 (1.25, 2.06)
**GASTRIC CANCER**			
**Cancer mortality**			
Non-smokers	Reference	Reference	Reference
Former smokers	1.04 (0.93, 1.16)	0.78 (0.65, 0.93)	1.14 (0.99, 1.31)
Light smoking quitters	1.40 (1.21, 1.61)	0.93 (0.73, 1.17)	1.58 (1.32, 1.88)
Heavy smoking quitters	1.51 (1.35, 1.69)	1.04 (0.86, 1.25)	1.68 (1.46, 1.95)
Consistent moderate smokers	1.75 (1.45, 2.11)	0.74 (0.52, 1.04)	2.19 (1.75, 2.74)
Decreasing heavy smokers	1.53 (1.22, 1.92)	0.94 (0.61, 1.46)	1.96 (1.50, 2.55)
**Cancer-specific mortality**			
Non-smokers	Reference	Reference	Reference
Former smokers	0.90 (0.77, 1.05)	0.74 (0.60, 0.92)	0.95 (0.76, 1.19)
Light smoking quitters	1.14 (0.94, 1.40)	0.77 (0.58, 1.03)	1.37 (1.03, 1.81)
Heavy smoking quitters	1.13 (0.96, 1.32)	0.94 (0.76, 1.17)	1.09 (0.86, 1.38)

Consistent moderate smokers	0.91 (0.67, 1.25)	0.57 (0.37, 0.88)	0.93 (0.58, 1.48)
Decreasing heavy smokers	0.84 (0.58, 1.22)	0.71 (0.41, 1.22)	1.00 (0.61, 1.65)
**COLORECTAL CANCER**			
**Cancer mortality**			
Non-smokers	Reference	Reference	Reference
Former smokers	1.08 (0.96, 1.21)	0.68 (0.57, 0.82)	1.21 (1.04, 1.41)
Light smoking quitters	1.23 (1.05, 1.45)	0.88 (0.68, 1.13)	1.33 (1.07, 1.66)
Heavy smoking quitters	1.34 (1.18, 1.53)	0.80 (0.65, 0.98)	1.58 (1.33, 1.86)
Consistent moderate smokers	1.36 (1.10, 1.70)	0.46 (0.28, 0.75)	1.94 (1.50, 2.49)
Decreasing heavy smokers	1.44 (1.10, 1.87)	0.99 (0.61, 1.62)	1.81 (1.31, 2.50)
**Cancer-specific mortality**			
Non-smokers	Reference	Reference	Reference
Former smokers	0.97 (0.84, 1.13)	0.63 (0.52, 0.78)	1.13 (0.91, 1.40)
Light smoking quitters	1.16 (0.95, 1.43)	0.87 (0.66, 1.15)	1.24 (0.91, 1.69)
Heavy smoking quitters	1.06 (0.90, 1.25)	0.66 (0.52, 0.83)	1.28 (1.01, 1.63)
Consistent moderate smokers	0.44 (0.28, 0.69)	0.25 (0.13, 0.50)	0.55 (0.31, 1.00)
Decreasing heavy smokers	0.76 (0.50, 1.14)	0.69 (0.38, 1.26)	0.84 (0.47, 1.49)
**LIVER CANCER**			
**Cancer mortality**			
Non-smokers	Reference	Reference	Reference
Former smokers	1.03 (0.92, 1.15)	0.81 (0.69, 0.97)	1.09 (0.94, 1.26)
Light smoking quitters	1.12 (0.96, 1.31)	0.92 (0.73, 1.17)	1.13 (0.93, 1.39)
Heavy smoking quitters	1.10 (0.98, 1.24)	0.94 (0.79, 1.12)	1.09 (0.93, 1.27)
Consistent moderate smokers	0.99 (0.80, 1.22)	0.88 (0.62, 1.24)	1.03 (0.79, 1.34)
Decreasing heavy smokers	1.03 (0.81, 1.30)	0.84 (0.53, 1.32)	1.23 (0.93, 1.63)
**Cancer-specific mortality**			
Non-smokers	Reference	Reference	Reference
Former smokers	1.02 (0.91, 1.15)	0.81 (0.68, 0.97)	1.09 (0.93, 1.27)
Light smoking quitters	1.06 (0.90, 1.26)	0.89 (0.69, 1.13)	1.05 (0.84, 1.31)
Heavy smoking quitters	1.06 (0.93, 1.20)	0.94 (0.77, 1.13)	1.01 (0.85, 1.21)
Consistent moderate smokers	0.90 (0.71, 1.13)	0.81 (0.55, 1.20)	0.92 (0.69, 1.23)
Decreasing heavy smokers	0.82 (0.62, 1.08)	0.78 (0.49, 1.24)	0.91 (0.65, 1.28)
**LUNG CANCER**			
**Cancer mortality**			
Non-smokers	Reference	Reference	Reference
Former smokers	0.96 (0.83, 1.11)	0.83 (0.68, 1.01)	1.05 (0.86, 1.28)
Light smoking quitters	1.07 (0.88, 1.29)	1.10 (0.85, 1.42)	1.10 (0.85, 1.42)
Heavy smoking quitters	1.04 (0.90, 1.19)	0.98 (0.80, 1.19)	1.12 (0.92, 1.35)
Consistent moderate smokers	1.18 (0.90, 1.55)	0.96 (0.62, 1.47)	1.34 (0.94, 1.92)
Decreasing heavy smokers	1.10 (0.82, 1.48)	0.81 (0.44, 1.50)	1.57 (1.10, 2.23)
**Cancer-specific mortality**			
Non-smokers	Reference	Reference	Reference

Former smokers	1.07 (0.90, 1.26)	0.84 (0.68, 1.05)	1.34 (1.04, 1.73)
Light smoking quitters	1.27 (1.03, 1.56)	1.16 (0.88, 1.52)	1.53 (1.12, 2.09)
Heavy smoking quitters	1.18 (1.00, 1.39)	0.99 (0.80, 1.23)	1.49 (1.17, 1.91)
Consistent moderate smokers	1.17 (0.84, 1.62)	0.91 (0.57, 1.46)	1.51 (0.95, 2.39)
Decreasing heavy smokers	0.95 (0.65, 1.39)	0.76 (0.40, 1.46)	1.47 (0.90, 2.41)

[a] adjusted hazard ratio, adjusted for age at cancer diagnosis, income, body mass index, alcohol drinking, physical activity, and Charlson Comorbidity Index;
[b] confidence interval.

Supplemental Table S10. All-cause mortality risk according to smoking trajectories with the decreasing heavy smokers as the reference

Smoking trajectories	aHR[a] (95% CI[b])		
	Overall	Subgroup: Overall survival <5 years	Subgroup: Overall survival ≥5 years
**ALL CANCERS**			
Non-smokers	0.68 (0.63, 0.74)	0.92 (0.77, 1.09)	0.57 (0.52, 0.63)
Former smokers	0.71 (0.65, 0.76)	0.72 (0.61, 0.85)	0.62 (0.57, 0.68)
Light smoking quitters	0.90 (0.83, 0.99)	1.01 (0.85, 1.21)	0.79 (0.71, 0.87)
Heavy smoking quitters	0.94 (0.87, 1.02)	0.97 (0.82, 1.15)	0.83 (0.76, 0.91)
Consistent moderate smokers	0.97 (0.88, 1.08)	0.88 (0.72, 1.08)	0.89 (0.79, 0.99)
Decreasing heavy smokers	Reference	Reference	Reference
**SMOKING-RELATED CANCERS**			
Non-smokers	0.69 (0.63, 0.76)	0.98 (0.81, 1.19)	0.57 (0.51, 0.63)
Former smokers	0.71 (0.65, 0.78)	0.74 (0.61, 0.90)	0.62 (0.56, 0.68)
Light smoking quitters	0.90 (0.82, 0.99)	1.01 (0.82, 1.23)	0.77 (0.69, 0.86)
Heavy smoking quitters	0.93 (0.85, 1.02)	0.96 (0.80, 1.17)	0.81 (0.73, 0.90)
Consistent moderate smokers	0.97 (0.86, 1.08)	0.87 (0.70, 1.10)	0.87 (0.77, 0.99)
Decreasing heavy smokers	Reference	Reference	Reference
**CANCERS UNRELATED TO SMOKING**			
Non-smokers	0.67 (0.56, 0.80)	0.80 (0.55, 1.16)	0.61 (0.49, 0.75)
Former smokers	0.70 (0.58, 0.84)	0.65 (0.45, 0.93)	0.65 (0.53, 0.80)
Light smoking quitters	0.91 (0.75, 1.11)	0.98 (0.66, 1.44)	0.85 (0.68, 1.07)
Heavy smoking quitters	0.96 (0.80, 1.15)	0.90 (0.62, 1.30)	0.89 (0.72, 1.11)
Consistent moderate smokers	1.01 (0.81, 1.26)	0.88 (0.57, 1.38)	0.96 (0.74, 1.23)
Decreasing heavy smokers	Reference	Reference	Reference
**GASTRIC CANCER**			
Non-smokers	0.58 (0.49, 0.68)	0.99 (0.67, 1.45)	0.48 (0.40, 0.58)
Former smokers	0.59 (0.50, 0.69)	0.75 (0.52, 1.10)	0.52 (0.44, 0.62)
Light smoking quitters	0.78 (0.66, 0.93)	1.01 (0.68, 1.50)	0.68 (0.56, 0.82)
Heavy smoking quitters	0.83 (0.70, 0.97)	1.01 (0.69, 1.46)	0.73 (0.61, 0.87)
Consistent moderate smokers	1.03 (0.85, 1.25)	1.07 (0.70, 1.65)	0.88 (0.71, 1.10)
Decreasing heavy smokers	Reference	Reference	Reference
**COLORECTAL CANCER**			
Non-smokers	0.64 (0.52, 0.79)	1.14 (0.73, 1.78)	0.52 (0.41, 0.67)
Former smokers	0.69 (0.56, 0.85)	0.83 (0.54, 1.29)	0.58 (0.46, 0.74)
Light smoking quitters	0.81 (0.64, 1.02)	1.15 (0.72, 1.83)	0.67 (0.52, 0.88)
Heavy smoking quitters	0.85 (0.68, 1.05)	0.95 (0.61, 1.48)	0.75 (0.58, 0.95)
Consistent moderate smokers	0.90 (0.69, 1.16)	0.99 (0.58, 1.69)	0.81 (0.60, 1.09)
Decreasing heavy smokers	Reference	Reference	Reference
**LIVER CANCER**			

Non-smokers	0.82 (0.66, 1.03)	1.04 (0.67, 1.61)	0.68 (0.52, 0.88)
Former smokers	0.88 (0.71, 1.09)	0.81 (0.52, 1.25)	0.78 (0.61, 1.00)
Light smoking quitters	0.99 (0.78, 1.24)	1.01 (0.64, 1.60)	0.81 (0.62, 1.07)
Heavy smoking quitters	0.97 (0.78, 1.20)	1.00 (0.65, 1.55)	0.80 (0.63, 1.03)
Consistent moderate smokers	0.81 (0.62, 1.07)	0.87 (0.51, 1.47)	0.72 (0.52, 0.98)
Decreasing heavy smokers	Reference	Reference	Reference
**LUNG CANCER**			
Non-smokers	0.82 (0.61, 1.10)	1.06 (0.58, 1.92)	0.59 (0.42, 0.83)
Former smokers	0.83 (0.63, 1.10)	0.94 (0.53, 1.69)	0.65 (0.47, 0.89)
Light smoking quitters	0.98 (0.72, 1.31)	1.22 (0.67, 2.24)	0.77 (0.54, 1.09)
Heavy smoking quitters	0.95 (0.72, 1.25)	1.13 (0.63, 2.01)	0.75 (0.55, 1.03)
Consistent moderate smokers	0.98 (0.69, 1.40)	1.03 (0.52, 2.04)	0.78 (0.51, 1.18)
Decreasing heavy smokers	Reference	Reference	Reference

[a] adjusted hazard ratio, adjusted for age at cancer diagnosis, income, body mass index, alcohol drinking, physical activity, and Charlson Comorbidity Index;
[b] confidence interval.

Supplemental Table S11. Mortality risks according to smoking trajectories for the entire study population with time-dependent covariates

**Pre-diagnosis smoking levels**	**aHR[a] (95% CI[b])**		
	**Overall**	**Subgroup: Overall survival <5 years**	**Subgroup: Overall survival ≥5 years**
**ALL CANCERS**			
**All-cause mortality**			
Non-smokers	Reference	Reference	Reference
Former smokers	1.04 (1.01, 1.08)	0.85 (0.80, 0.91)	1.07 (1.03, 1.12)
Light smoking quitters	1.33 (1.27, 1.40)	1.03 (0.94, 1.12)	1.37 (1.29, 1.45)
Heavy smoking quitters	1.41 (1.36, 1.47)	1.03 (0.96, 1.11)	1.45 (1.39, 1.52)
Consistent moderate smokers	1.43 (1.34, 1.54)	0.88 (0.76, 1.01)	1.52 (1.40, 1.65)
Decreasing heavy smokers	1.50 (1.38, 1.63)	1.05 (0.88, 1.26)	1.73 (1.58, 1.90)
**Cancer mortality**			
Non-smokers	Reference	Reference	Reference
Former smokers	1.06 (1.01, 1.11)	0.85 (0.79, 0.92)	1.10 (1.04, 1.16)
Light smoking quitters	1.38 (1.30, 1.47)	1.02 (0.92, 1.13)	1.45 (1.35, 1.57)
Heavy smoking quitters	1.46 (1.40, 1.54)	1.05 (0.97, 1.14)	1.53 (1.44, 1.62)
Consistent moderate smokers	1.45 (1.33, 1.58)	0.78 (0.66, 0.92)	1.64 (1.48, 1.82)
Decreasing heavy smokers	1.44 (1.30, 1.59)	1.05 (0.86, 1.27)	1.71 (1.52, 1.92)
**GASTRIC CANCER**			
**All-cause mortality**			
Non-smokers	Reference	Reference	Reference
Former smokers	1.02 (0.95, 1.11)	0.80 (0.68, 0.94)	1.07 (0.98, 1.17)
Light smoking quitters	1.36 (1.23, 1.51)	0.99 (0.81, 1.21)	1.41 (1.25, 1.58)
Heavy smoking quitters	1.44 (1.32, 1.56)	0.97 (0.83, 1.14)	1.49 (1.36, 1.64)
Consistent moderate smokers	1.74 (1.51, 2.00)	0.99 (0.75, 1.29)	1.75 (1.49, 2.07)
Decreasing heavy smokers	1.72 (1.45, 2.03)	0.95 (0.64, 1.40)	2.00 (1.67, 2.41)
**Cancer mortality**			
Non-smokers	Reference	Reference	Reference
Former smokers	1.06 (0.95, 1.18)	0.80 (0.66, 0.97)	1.16 (1.01, 1.33)
Light smoking quitters	1.47 (1.28, 1.70)	0.93 (0.72, 1.19)	1.66 (1.39, 1.97)
Heavy smoking quitters	1.59 (1.42, 1.79)	1.02 (0.84, 1.23)	1.73 (1.51, 2.00)
Consistent moderate smokers	1.83 (1.51, 2.21)	0.73 (0.51, 1.05)	2.19 (1.74, 2.74)
Decreasing heavy smokers	1.63 (1.30, 2.05)	0.94 (0.60, 1.47)	2.03 (1.56, 2.65)
**Cancer-specific mortality**			
Non-smokers	Reference	Reference	Reference
Former smokers	0.91 (0.78, 1.07)	0.75 (0.60, 0.94)	0.99 (0.79, 1.23)
Light smoking quitters	1.23 (1.01, 1.51)	0.78 (0.58, 1.06)	1.52 (1.16, 2.00)
Heavy smoking quitters	1.21 (1.03, 1.42)	0.95 (0.76, 1.18)	1.15 (0.91, 1.44)
Consistent moderate smokers	0.91 (0.66, 1.27)	0.56 (0.36, 0.87)	0.89 (0.55, 1.42)
Decreasing heavy smokers	0.90 (0.62, 1.31)	0.72 (0.41, 1.26)	1.04 (0.63, 1.71)

**COLORECTAL CANCER**			
**All-cause mortality**			
Non-smokers	Reference	Reference	Reference
Former smokers	1.08 (0.98, 1.18)	0.82 (0.69, 0.98)	1.10 (0.98, 1.22)
Light smoking quitters	1.22 (1.07, 1.39)	0.87 (0.69, 1.11)	1.24 (1.07, 1.45)
Heavy smoking quitters	1.37 (1.23, 1.52)	0.87 (0.72, 1.06)	1.42 (1.26, 1.61)
Consistent moderate smokers	1.36 (1.13, 1.63)	0.78 (0.53, 1.15)	1.47 (1.19, 1.82)
Decreasing heavy smokers	1.61 (1.29, 2.00)	1.02 (0.64, 1.63)	1.89 (1.48, 2.42)
**Cancer mortality**			
Non-smokers	Reference	Reference	Reference
Former smokers	1.11 (0.99, 1.25)	0.77 (0.63, 0.93)	1.20 (1.03, 1.39)
Light smoking quitters	1.22 (1.03, 1.45)	0.78 (0.59, 1.03)	1.33 (1.07, 1.64)
Heavy smoking quitters	1.45 (1.28, 1.66)	0.86 (0.69, 1.06)	1.59 (1.35, 1.88)
Consistent moderate smokers	1.41 (1.12, 1.77)	0.49 (0.29, 0.82)	1.84 (1.42, 2.39)
Decreasing heavy smokers	1.53 (1.16, 2.00)	1.08 (0.66, 1.77)	1.83 (1.32, 2.54)
**Cancer-specific mortality**			
Non-smokers	Reference	Reference	Reference
Former smokers	1.01 (0.87, 1.18)	0.74 (0.60, 0.92)	1.09 (0.88, 1.34)
Light smoking quitters	1.09 (0.88, 1.36)	0.76 (0.56, 1.04)	1.15 (0.84, 1.57)
Heavy smoking quitters	1.17 (0.99, 1.39)	0.74 (0.59, 0.95)	1.28 (1.01, 1.62)
Consistent moderate smokers	0.47 (0.30, 0.74)	0.27 (0.13, 0.56)	0.54 (0.30, 0.98)
Decreasing heavy smokers	0.76 (0.49, 1.16)	0.74 (0.40, 1.38)	0.79 (0.44, 1.44)
**LIVER CANCER**			
**All-cause mortality**			
Non-smokers	Reference	Reference	Reference
Former smokers	1.12 (1.00, 1.24)	0.89 (0.74, 1.06)	1.19 (1.04, 1.36)
Light smoking quitters	1.24 (1.08, 1.43)	0.95 (0.75, 1.21)	1.26 (1.06, 1.51)
Heavy smoking quitters	1.23 (1.10, 1.37)	0.93 (0.77, 1.12)	1.22 (1.06, 1.41)
Consistent moderate smokers	0.99 (0.81, 1.21)	0.71 (0.49, 1.02)	1.06 (0.83, 1.35)
Decreasing heavy smokers	1.31 (1.05, 1.63)	0.93 (0.60, 1.46)	1.55 (1.20, 2.00)
**Cancer mortality**			
Non-smokers	Reference	Reference	Reference
Former smokers	1.10 (0.98, 1.23)	0.91 (0.76, 1.10)	1.15 (0.99, 1.33)
Light smoking quitters	1.20 (1.03, 1.40)	0.92 (0.72, 1.18)	1.21 (1.00, 1.48)
Heavy smoking quitters	1.20 (1.06, 1.35)	0.93 (0.76, 1.12)	1.17 (1.00, 1.37)
Consistent moderate smokers	0.99 (0.79, 1.22)	0.73 (0.50, 1.06)	1.05 (0.80, 1.36)
Decreasing heavy smokers	1.20 (0.94, 1.52)	0.86 (0.53, 1.40)	1.42 (1.07, 1.89)
**Cancer-specific mortality**			
Non-smokers	Reference	Reference	Reference
Former smokers	1.09 (0.96, 1.23)	0.89 (0.73, 1.09)	1.15 (0.98, 1.35)
Light smoking quitters	1.15 (0.97, 1.36)	0.89 (0.68, 1.15)	1.16 (0.93, 1.44)
Heavy smoking quitters	1.15 (1.01, 1.31)	0.89 (0.73, 1.10)	1.11 (0.94, 1.32)

Consistent moderate smokers	0.91 (0.71, 1.15)	0.68 (0.45, 1.02)	0.96 (0.72, 1.30)
Decreasing heavy smokers	0.94 (0.71, 1.26)	0.81 (0.48, 1.36)	1.08 (0.77, 1.52)
**LUNG CANCER**			
**All-cause mortality**			
Non-smokers	Reference	Reference	Reference
Former smokers	1.02 (0.90, 1.17)	1.01 (0.82, 1.24)	1.10 (0.93, 1.31)
Light smoking quitters	1.26 (1.06, 1.49)	1.27 (0.98, 1.66)	1.34 (1.08, 1.67)
Heavy smoking quitters	1.21 (1.07, 1.38)	1.13 (0.92, 1.39)	1.36 (1.16, 1.61)
Consistent moderate smokers	1.18 (0.91, 1.53)	0.88 (0.56, 1.38)	1.33 (0.97, 1.83)
Decreasing heavy smokers	1.23 (0.91, 1.65)	0.90 (0.47, 1.73)	1.72 (1.22, 2.41)
**Cancer mortality**			
Non-smokers	Reference	Reference	Reference
Former smokers	0.97 (0.84, 1.13)	0.96 (0.77, 1.20)	1.06 (0.87, 1.30)
Light smoking quitters	1.18 (0.98, 1.42)	1.24 (0.94, 1.64)	1.23 (0.95, 1.60)
Heavy smoking quitters	1.11 (0.97, 1.28)	1.06 (0.86, 1.32)	1.25 (1.03, 1.51)
Consistent moderate smokers	1.14 (0.86, 1.52)	0.81 (0.50, 1.31)	1.34 (0.93, 1.93)
Decreasing heavy smokers	1.15 (0.83, 1.60)	0.86 (0.43, 1.72)	1.67 (1.15, 2.45)
**Cancer-specific mortality**			
Non-smokers	Reference	Reference	Reference
Former smokers	1.07 (0.90, 1.27)	0.98 (0.78, 1.24)	1.32 (1.02, 1.69)
Light smoking quitters	1.36 (1.10, 1.68)	1.31 (0.98, 1.75)	1.62 (1.19, 2.22)
Heavy smoking quitters	1.25 (1.06, 1.47)	1.09 (0.87, 1.36)	1.62 (1.27, 2.07)
Consistent moderate smokers	1.09 (0.77, 1.54)	0.73 (0.43, 1.24)	1.45 (0.91, 2.31)
Decreasing heavy smokers	0.99 (0.66, 1.48)	0.85 (0.41, 1.75)	1.57 (0.95, 2.59)

[a] adjusted hazard ratio, adjusted for age at cancer diagnosis, income, body mass index, alcohol drinking, physical activity, and Charlson Comorbidity Index;
[b] confidence interval.

Supplemental Table S12. Mortality risks according to smoking trajectories for the three-measurement subpopulation with time-dependent covariates

Smoking trajectories	aHR[a] (95% CI[b])		
	Overall	Subgroup: Overall survival <5 years	Subgroup: Overall survival ≥5 years
**ALL CANCERS**			
**All-cause mortality**			
Non-smokers	Reference	Reference	Reference
Former smokers	1.11 (1.03, 1.19)	0.95 (0.78, 1.14)	1.14 (1.06, 1.23)
Light smoking quitters	1.39 (1.27, 1.53)	0.83 (0.64, 1.08)	1.45 (1.31, 1.60)
Heavy smoking quitters	1.52 (1.41, 1.65)	1.06 (0.86, 1.30)	1.56 (1.44, 1.70)
Late heavy-smoking quitters	1.67 (1.46, 1.91)	1.04 (0.69, 1.56)	1.77 (1.54, 2.04)
Heavy smoking relapse	1.65 (1.47, 1.87)	0.80 (0.57, 1.11)	1.74 (1.53, 1.98)
Consistent heavy smokers	1.62 (1.43, 1.85)	0.85 (0.58, 1.24)	1.73 (1.51, 1.98)
**Cancer mortality**			
Non-smokers	Reference	Reference	Reference
Former smokers	1.13 (1.03, 1.24)	0.88 (0.71, 1.09)	1.19 (1.08, 1.31)
Light smoking quitters	1.52 (1.35, 1.71)	0.90 (0.67, 1.21)	1.59 (1.40, 1.82)
Heavy smoking quitters	1.60 (1.45, 1.76)	1.11 (0.88, 1.40)	1.66 (1.49, 1.85)
Late heavy-smoking quitters	1.71 (1.45, 2.03)	1.03 (0.65, 1.64)	1.86 (1.55, 2.23)
Heavy smoking relapse	1.67 (1.43, 1.94)	0.67 (0.45, 1.00)	1.84 (1.56, 2.17)
Consistent heavy smokers	1.78 (1.52, 2.07)	0.82 (0.54, 1.26)	1.96 (1.66, 2.31)
**GASTRIC CANCER**			
**All-cause mortality**			
Non-smokers	Reference	Reference	Reference
Former smokers	1.20 (1.04, 1.38)	0.82 (0.53, 1.25)	1.26 (1.08, 1.48)
Light smoking quitters	1.58 (1.30, 1.91)	0.77 (0.43, 1.38)	1.67 (1.36, 2.05)
Heavy smoking quitters	1.80 (1.53, 2.11)	0.82 (0.53, 1.28)	1.87 (1.57, 2.21)
Late heavy-smoking quitters	1.87 (1.40, 2.49)	0.30 (0.09, 1.02)	2.13 (1.58, 2.87)
Heavy smoking relapse	1.98 (1.57, 2.49)	0.61 (0.28, 1.34)	2.20 (1.73, 2.80)
Consistent heavy smokers	1.99 (1.52, 2.60)	0.53 (0.20, 1.44)	2.26 (1.70, 2.99)
**Cancer mortality**			
Non-smokers	Reference	Reference	Reference
Former smokers	1.24 (0.99, 1.55)	0.86 (0.48, 1.54)	1.35 (1.05, 1.72)
Light smoking quitters	1.87 (1.41, 2.48)	1.07 (0.51, 2.25)	2.01 (1.48, 2.74)
Heavy smoking quitters	2.20 (1.74, 2.77)	1.24 (0.70, 2.21)	2.28 (1.76, 2.96)
Late heavy-smoking quitters	2.57 (1.75, 3.79)	0.55 (0.15, 2.00)	3.01 (2.00, 4.54)
Heavy smoking relapse	2.26 (1.62, 3.15)	0.38 (0.11, 1.35)	2.79 (1.96, 3.96)
Consistent heavy smokers	2.44 (1.69, 3.53)	0.59 (0.16, 2.13)	2.97 (2.01, 4.40)
**Cancer-specific mortality**			
Non-smokers	Reference	Reference	Reference
Former smokers	1.06 (0.76, 1.48)	0.95 (0.47, 1.92)	1.13 (0.76, 1.66)

Light smoking quitters	1.47 (0.95, 2.28)	0.77 (0.28, 2.12)	1.72 (1.05, 2.82)
Heavy smoking quitters	1.58 (1.11, 2.24)	1.34 (0.67, 2.68)	1.43 (0.93, 2.19)
Late heavy-smoking quitters	0.44 (0.14, 1.41)	0.26 (0.03, 2.08)	0.43 (0.10, 1.79)
Heavy smoking relapse	1.69 (1.01, 2.83)	0.38 (0.08, 1.75)	2.29 (1.31, 4.00)
Consistent heavy smokers	0.74 (0.33, 1.64)	0.63 (0.13, 3.01)	0.78 (0.30, 1.99)
**COLORECTAL CANCER**			
**All-cause mortality**			
Non-smokers	Reference	Reference	Reference
Former smokers	1.18 (0.99, 1.42)	1.31 (0.77, 2.23)	1.16 (0.96, 1.41)
Light smoking quitters	1.47 (1.14, 1.89)	0.67 (0.28, 1.61)	1.61 (1.23, 2.10)
Heavy smoking quitters	1.52 (1.23, 1.89)	1.35 (0.73, 2.47)	1.51 (1.20, 1.91)
Late heavy-smoking quitters	1.56 (1.07, 2.29)	5.35 (1.45, 19.77)	1.67 (1.12, 2.50)
Heavy smoking relapse	1.85 (1.35, 2.51)	1.09 (0.38, 3.11)	2.02 (1.46, 2.79)
Consistent heavy smokers	2.02 (1.48, 2.76)	1.92 (0.86, 4.28)	1.96 (1.39, 2.77)
**Cancer mortality**			
Non-smokers	Reference	Reference	Reference
Former smokers	1.33 (1.04, 1.70)	1.28 (0.69, 2.38)	1.31 (1.00, 1.73)
Light smoking quitters	1.66 (1.18, 2.33)	1.02 (0.40, 2.58)	1.83 (1.26, 2.64)
Heavy smoking quitters	1.55 (1.16, 2.08)	1.35 (0.67, 2.74)	1.53 (1.11, 2.12)
Late heavy-smoking quitters	1.68 (1.02, 2.76)	3.77 (0.79, 18.01)	1.88 (1.11, 3.21)
Heavy smoking relapse	2.09 (1.41, 3.11)	1.21 (0.37, 3.95)	2.36 (1.54, 3.61)
Consistent heavy smokers	2.61 (1.79, 3.79)	2.09 (0.85, 5.16)	2.66 (1.75, 4.04)
**Cancer-specific mortality**			
Non-smokers	Reference	Reference	Reference
Former smokers	1.10 (0.79, 1.54)	1.27 (0.62, 2.59)	1.01 (0.68, 1.48)
Light smoking quitters	1.16 (0.71, 1.89)	1.31 (0.49, 3.51)	1.12 (0.63, 1.98)
Heavy smoking quitters	1.29 (0.88, 1.88)	1.23 (0.55, 2.76)	1.18 (0.75, 1.84)
Late heavy-smoking quitters	1.09 (0.51, 2.31)	-	1.44 (0.67, 3.11)
Heavy smoking relapse	0.79 (0.39, 1.62)	1.07 (0.27, 4.23)	0.71 (0.30, 1.69)
Consistent heavy smokers	0.77 (0.38, 1.59)	1.10 (0.32, 3.74)	0.59 (0.23, 1.51)
**LIVER CANCER**			
**All-cause mortality**			
Non-smokers	Reference	Reference	Reference
Former smokers	1.03 (0.84, 1.25)	1.40 (0.85, 2.31)	1.05 (0.84, 1.31)
Light smoking quitters	1.11 (0.85, 1.46)	1.22 (0.66, 2.27)	1.08 (0.79, 1.47)
Heavy smoking quitters	1.08 (0.86, 1.36)	1.46 (0.86, 2.47)	1.03 (0.79, 1.33)
Late heavy-smoking quitters	1.03 (0.69, 1.53)	1.54 (0.62, 3.81)	0.93 (0.59, 1.47)
Heavy smoking relapse	1.21 (0.87, 1.70)	1.97 (0.81, 4.78)	1.29 (0.89, 1.87)
Consistent heavy smokers	1.12 (0.79, 1.59)	1.07 (0.41, 2.80)	1.21 (0.83, 1.79)
**Cancer mortality**			
Non-smokers	Reference	Reference	Reference
Former smokers	1.06 (0.85, 1.31)	1.49 (0.89, 2.49)	1.08 (0.84, 1.38)

Light smoking quitters	1.11 (0.83, 1.50)	1.27 (0.67, 2.40)	1.08 (0.77, 1.52)
Heavy smoking quitters	1.07 (0.84, 1.36)	1.52 (0.88, 2.62)	1.00 (0.75, 1.32)
Late heavy-smoking quitters	1.01 (0.65, 1.55)	1.23 (0.43, 3.45)	0.98 (0.60, 1.60)
Heavy smoking relapse	1.17 (0.81, 1.68)	1.81 (0.70, 4.67)	1.27 (0.85, 1.91)
Consistent heavy smokers	1.09 (0.74, 1.60)	0.91 (0.32, 2.57)	1.22 (0.81, 1.86)
**Cancer-specific mortality**			
Non-smokers	Reference	Reference	Reference
Former smokers	1.08 (0.85, 1.36)	1.52 (0.89, 2.58)	1.11 (0.84, 1.45)
Light smoking quitters	1.11 (0.81, 1.54)	1.28 (0.66, 2.48)	1.07 (0.73, 1.57)
Heavy smoking quitters	1.05 (0.81, 1.37)	1.43 (0.81, 2.53)	1.00 (0.74, 1.37)
Late heavy-smoking quitters	0.83 (0.50, 1.40)	0.85 (0.24, 3.02)	0.87 (0.49, 1.55)
Heavy smoking relapse	1.21 (0.82, 1.80)	1.95 (0.75, 5.07)	1.34 (0.86, 2.08)
Consistent heavy smokers	0.89 (0.57, 1.39)	1.02 (0.36, 2.90)	0.96 (0.58, 1.59)
**LUNG CANCER**			
**All-cause mortality**			
Non-smokers	Reference	Reference	Reference
Former smokers	0.93 (0.71, 1.21)	2.18 (1.20, 3.94)	1.08 (0.79, 1.47)
Light smoking quitters	1.23 (0.87, 1.73)	0.48 (0.18, 1.26)	1.45 (0.99, 2.14)
Heavy smoking quitters	1.19 (0.92, 1.54)	1.38 (0.75, 2.55)	1.47 (1.09, 1.98)
Late heavy-smoking quitters	1.66 (1.02, 2.71)	1.67 (0.27, 10.28)	2.08 (1.22, 3.54)
Heavy smoking relapse	1.52 (0.96, 2.40)	2.14 (0.86, 5.32)	1.34 (0.77, 2.35)
Consistent heavy smokers	1.02 (0.61, 1.71)	0.85 (0.18, 4.14)	1.21 (0.69, 2.13)
**Cancer mortality**			
Non-smokers	Reference	Reference	Reference
Former smokers	0.77 (0.57, 1.04)	1.83 (0.97, 3.44)	0.90 (0.63, 1.27)
Light smoking quitters	1.07 (0.73, 1.57)	0.48 (0.17, 1.32)	1.25 (0.81, 1.94)
Heavy smoking quitters	1.03 (0.78, 1.37)	1.27 (0.66, 2.45)	1.29 (0.93, 1.80)
Late heavy-smoking quitters	1.52 (0.88, 2.61)	1.80 (0.27, 12.00)	1.87 (1.04, 3.39)
Heavy smoking relapse	1.43 (0.86, 2.37)	1.79 (0.66, 4.84)	1.30 (0.70, 2.42)
Consistent heavy smokers	0.89 (0.50, 1.60)	0.45 (0.05, 3.74)	1.13 (0.61, 2.11)
**Cancer-specific mortality**			
Non-smokers	Reference	Reference	Reference
Former smokers	0.89 (0.62, 1.27)	2.43 (1.22, 4.86)	1.02 (0.66, 1.58)
Light smoking quitters	1.26 (0.80, 1.97)	0.53 (0.18, 1.58)	1.51 (0.89, 2.57)
Heavy smoking quitters	1.26 (0.90, 1.77)	1.60 (0.77, 3.30)	1.65 (1.10, 2.50)
Late heavy-smoking quitters	1.38 (0.69, 2.76)	1.41 (0.12, 15.92)	1.82 (0.85, 3.89)
Heavy smoking relapse	1.77 (0.99, 3.15)	2.70 (0.95, 7.70)	1.47 (0.69, 3.13)
Consistent heavy smokers	0.58 (0.25, 1.37)	2.43 (1.22, 4.86)	0.84 (0.35, 2.04)

[a] adjusted hazard ratio, adjusted for age at cancer diagnosis, income, body mass index, alcohol drinking, physical activity, and Charlson Comorbidity Index;
[b] confidence interval.

Supplemental Table S13. Model details and evaluation for smoking trajectories of pre-diagnosis current smokers with three to six post-diagnosis smoking measurements

**Group**	**n**[a]	**p**[b]	**π**[c]	**AvePP**[d]	**OCC**[e]	**Consistent post-diagnosis smoking behavior,** n (%)
**Subgroup of three post-diagnosis measurements** (n=24340, 53.7% of pre-diagnosis current smokers)-Model A (2,2,2,2)						
Consistent quitters	15641	64.3	65.0	1.00	150.1	18604 (76.4)
Relapse	3463	14.2	13.5	0.92	75.7	
Late quitters	2273	9.3	9.1	0.90	87.7	
Consistent heavy smokers	2963	12.2	12.5	0.98	354.8	
**Subgroup of four post-diagnosis measurements** (n=16257, 35.9% of pre-diagnosis current smokers)-Model B (2,2,2,2,2)						
Consistent quitters	10742	66.1	65.7	0.99	40.5	12667 (77.9)
Relapse	1301	8.0	9.2	0.96	216.3	
Late quitters	2289	14.1	13.1	0.87	43.5	
Consistent heavy smokers	1925	11.8	12.0	0.87	48.1	
**Subgroup of five post-diagnosis measurements** (n=10012, 22.1% of pre-diagnosis current smokers)-Model C (2,2,2)						
Consistent quitters	6689	66.8	66.8	0.99	39.1	8164 (81.5)
Decreasing moderate smokers	1848	18.5	18.6	0.92	49.6	
Consistent heavy smokers	1475	14.7	14.6	0.95	107.5	
**Subgroup of six post-diagnosis measurements** (n=5871, 13.0% of pre-diagnosis current smokers)-Model D (2,2,2,2,2)						
Consistent quitters	3779	64.4	64.3	0.99	53.9	4441 (75.6)
Relapse-moderate smokers	609	10.4	10.2	0.87	60.8	
Relapse-heavy smokers	299	5.1	5.0	0.87	130.1	
Late quitters	522	8.9	9.1	0.93	139.0	
Consistent heavy smokers	662	11.3	11.4	0.96	196.9	

[a] number of participants;
[b] percentage of participants assigned to the smoking trajectory group (%);
[c] estimated group membership of the smoking trajectory (%);
[d] average posterior probability;
[e] odds of correct classification.

© 2023 Bui T.T. et al.
